# Linking Proteomic and Transcriptional Data through the Interactome and Epigenome Reveals a Map of Oncogene-induced Signaling

**DOI:** 10.1371/journal.pcbi.1002887

**Published:** 2013-02-07

**Authors:** Shao-shan Carol Huang, David C. Clarke, Sara J. C. Gosline, Adam Labadorf, Candace R. Chouinard, William Gordon, Douglas A. Lauffenburger, Ernest Fraenkel

**Affiliations:** 1 PhD Program in Computational and Systems Biology, Massachusetts Institute of Technology, Cambridge, Massachusetts, United States of America; 2 Department of Biological Engineering, Massachusetts Institute of Technology, Cambridge, Massachusetts, United States of America; 3 Center for Cellular Decision Processes, Massachusetts Institute of Technology, Cambridge, Massachusetts, United States of America; 4 Computer Science and Artificial Intelligence Laboratory, Massachusetts Institute of Technology, Cambridge, Massachusetts, United States of America; University of Washington, United States of America

## Abstract

Cellular signal transduction generally involves cascades of post-translational protein modifications that rapidly catalyze changes in protein-DNA interactions and gene expression. High-throughput measurements are improving our ability to study each of these stages individually, but do not capture the connections between them. Here we present an approach for building a network of physical links among these data that can be used to prioritize targets for pharmacological intervention. Our method recovers the critical missing links between proteomic and transcriptional data by relating changes in chromatin accessibility to changes in expression and then uses these links to connect proteomic and transcriptome data. We applied our approach to integrate epigenomic, phosphoproteomic and transcriptome changes induced by the variant III mutation of the epidermal growth factor receptor (EGFRvIII) in a cell line model of glioblastoma multiforme (GBM). To test the relevance of the network, we used small molecules to target highly connected nodes implicated by the network model that were not detected by the experimental data in isolation and we found that a large fraction of these agents alter cell viability. Among these are two compounds, ICG-001, targeting CREB binding protein (CREBBP), and PKF118–310, targeting β-catenin (CTNNB1), which have not been tested previously for effectiveness against GBM. At the level of transcriptional regulation, we used chromatin immunoprecipitation sequencing (ChIP-Seq) to experimentally determine the genome-wide binding locations of p300, a transcriptional co-regulator highly connected in the network. Analysis of p300 target genes suggested its role in tumorigenesis. We propose that this general method, in which experimental measurements are used as constraints for building regulatory networks from the interactome while taking into account noise and missing data, should be applicable to a wide range of high-throughput datasets.

## Author Summary

The ways in which cells respond to changes in their environment are controlled by networks of physical links among the proteins and genes. The initial signal of a change in conditions rapidly passes through these networks from the cytoplasm to the nucleus, where it can lead to long-term alterations in cellular behavior by controlling the expression of genes. These cascades of signaling events underlie many normal biological processes. As a result, being able to map out how these networks change in disease can provide critical insights for new approaches to treatment. We present a computational method for reconstructing these networks by finding links between the rapid short-term changes in proteins and the longer-term changes in gene regulation. This method brings together systematic measurements of protein signaling, genome organization and transcription in the context of protein-protein and protein-DNA interactions. When used to analyze datasets from an oncogene expressing cell line model of human glioblastoma, our approach identifies key nodes that affect cell survival and functional transcriptional regulators.

## Introduction

Cellular signaling and transcription are tightly integrated processes that underlie many short- and long-term cellular responses to the environment. Dysregulation of these molecular events has been implicated in diverse diseases including neurodegeneration [1], [2], metabolic disorders [3], and every stage of tumor development and growth [4], [5]. Sophisticated algorithms have been developed to use transcription profiling data for discovery of regulatory networks in disease, either *de novo* or from an interactome network (see review of theory [6] and tools [7]). Despite the utility of these methods, they suffer from the limitation that they use gene transcripts as a proxy for proteomic changes. As a result, they are unable to capture post-transcriptional changes in proteins, which are an important part of signaling and regulation.

The advent of improved proteomic methods has the potential to provide a systematic map of critical signaling pathways that are altered in disease. Computational approaches to combine transcriptional and proteomic data have focused on assessing the correlation between the data sources [8]–[10]. Some network analyses of proteomic and transcriptional data treated both as evidence of changes in protein levels, which were then viewed in the context of known pathway models [11], [12]. By equating transcripts and the proteins they encode, such network models do not make full use of the data. Alternative approaches that treat proteomic and transcriptional data as distinct can examine how proteomic signaling drives changes in gene regulation. Methods that search for physical associations among proteins and between proteome and the genome are likely to be particularly important in the analysis of phosphoproteomic data from mass spectrometry. Phosphoproteomics selectively measures protein phosphorylation, a principal biochemical mechanism of cellular signaling controlling gene expression. Since changes in phosphorylation and transcription are poorly correlated ([13] and Figure S1), they are highly complementary, providing distinct windows into cellular processes.

Previously, we have shown that phosphoproteomic and transcriptional data from the yeast *Saccharomyces cerevisiae* pheromone response could be linked through physical networks in a framework of constraint optimization on interactome networks known as the prize-collecting Steiner tree (PCST) [14]. This approach revealed relevant proteins and pathways that could not be discovered when each type of data was analyzed in isolation. Furthermore, it identified a network more compact and functionally relevant than networks constructed from direct interactors of the phosphoproteomic hits and transcription factors or by a related network optimization method ResponseNet [15], [16]. However, the method depended on the availability of experimentally determined genome-wide binding locations for almost all the transcriptional regulators in yeast [17], [18]. Such data are unavailable for mammalian cells in which the most comprehensive analysis so far has produced data for fewer than 10% of the transcription factors and only in a limited number of cell types [19].

**Figure 1:**
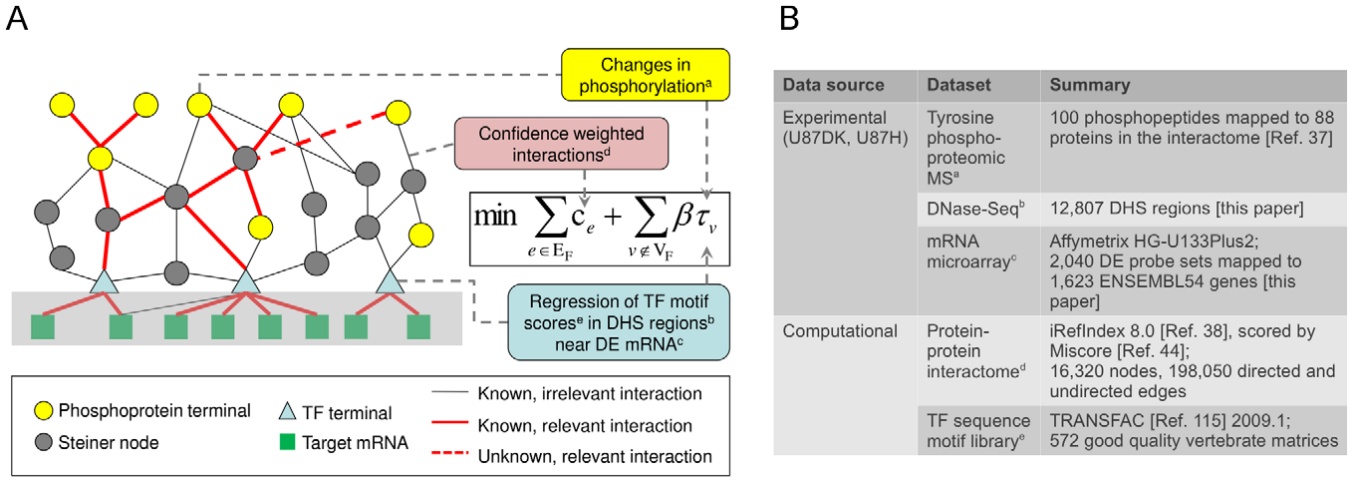
Setting up the PCST problem. A. Finding a network of interactions that link phosphorylation events and differentially transcribed genes can be formulated as an optimization problem on a protein interactome. The objective function (equation in box) represents a balance between excluding nodes for which there is experimental evidence (phosphorylated proteins as yellow circles and transcription factors as blue triangles) and including edges weighted by reliability. The light grey rectangle containing edges from transcription factors to target mRNAs indicates these edges are not directly included in the interactome. Instead, they are used to infer the activity of transcription factor candidates (see Materials and Methods). The optimal solution to the PCST problem connects the phoshoprotein termini and the transcription factor termini by reliable interactions (red lines) that may involve nodes not explicitly observed in the experimental data (Steiner nodes; dark grey circles). TF: transcription factor. DHS: differentially hypersensitive. DE: differential expression. The superscripts a to e correspond to the superscript labels of input data types in B. B. The input datasets from U87MG EGFRvIII-expressing cells used in this study.

In order to study disease-related pathways in mammalian cells, we developed a combined computational and experimental strategy that predicts transcription factors with altered binding or activity relevant to a particular cell type under specific conditions. First, we used DNaseI-hypersensitivity site sequencing (DNase-Seq) [20] to identify regions bound by as yet unknown regulatory proteins in each condition of interest. Scanning these sequences with a library of motifs revealed preliminary candidates of relevant binding proteins [21]–[23]. To discover the subset of these motifs most likely to be regulatory, we employed a regression-based method to infer the activity of specific transcription factors [24]–[26]. For each potential regulatory protein, we tested whether the quality of transcription factor motif matches in differentially hypersensitive regions correlated with the expression level of nearby genes. We then searched for protein-protein interactions that link these transcriptional regulators to upstream phosphoproteomic events by solving a PCST problem on the interactome (Figure 1A).

Beginning with an interactome network in which the reliability of each interaction is weighted by experimental evidence, we find an optimal subnetwork of the most reliable interactions that include a subset of the phosphorylation events and the transcriptional regulators, with preferences given to phosphorylation events that undergo large changes and transcriptional regulators that show strong activities. An important aspect of the PCST algorithm is that it is able to naturally account for missing data and false positives. In particular, since the input experimental data do not capture every relevant protein, we allow the network to include proteins that were not explicitly measured in the proteomic or transcriptomic assays. In addition, to account for false positives in the data, we allow the algorithm to exclude experimentally determined proteins and genes that are not connected with high confidence interactions. As a result, the final networks are compact, enriched for functionally relevant proteins and the most reliable interactions that include these proteins, and can be used to guide subsequent experiments. To further account for possible noise in the input datasets, we merge an optimal PCST solution with a set of suboptimal solutions to obtain a robust final network (see Materials and Methods).

Here we apply our integrated approach to prioritize experiments for probing the signaling pathways downstream of a mutant epidermal growth factor receptor (EGFR) in glioblastoma multiforme (GBM). The variant III mutant of EGFR (EGFRvIII) is the most common deletion mutant of EGFR in human cancer [27] and its levels are highly correlated with poor prognosis in GBM [28]–[30]. The deletion of exons 2–7 removes most of the extracellular ligand binding domain, so it is unable to bind EGF or other EGFR-binding ligands [31]. Nevertheless, the mutant receptor is constitutively phosphorylated [32], and is capable of activating downstream signaling pathways at a low level. Unlike wild-type EGFR signaling, which shuts itself off through a process known as receptor-mediated down-regulation, EGFRvIII signaling does not, leading to its oncogenic properties [31]. To comprehensively identify the downstream signaling consequences of the EGFRvIII, we incorporated phosphoproteomic, transcription profiling and DNase-Seq data from U87MG glioblastoma cells expressing this oncogenic mutant receptor (Figure 1B). In addition to recapitulating many known components in EGFRvIII signaling and transcriptional regulation, our network predicts key signaling nodes not apparent from the experimental data and provides a method for prioritizing experimental tests. We validated several of these predictions through pharmacological tests and genome-wide protein-DNA binding measurements. We propose that combining epigenomic methods to uncover transcriptional regulators and constraint optimization on biological networks effectively organizes disparate transcriptional and proteomic data, and can be used to discover unknown components of biological responses leading, potentially, to new therapeutic strategies.

## Results

To understand the signaling pathways downstream of EGFRvIII, we used as a model two cell lines derived from human U87MG glioblastoma cells that were engineered to express high levels of EGFRvIII (U87H; 2 million receptors per cell) or a mutant form of the receptor with an inactive kinase (U87DK) [31], [32] (Figure 1B). U87MG is a widely used cell line model for human grade IV glioma and many aspects of its biology have been well characterized. Independent long-term cultures of these cells are genetically stable [33], making it possible to interpret results from different laboratories at different times. Many findings from EGFRvIII expressing-U87MG cells have been validated *in vivo* [31], [32], [34]–[37]. Since the signaling network for wild-type EGFR is well established but not for EGFRvIII, this system provided us the opportunity to explore the global biochemical events downstream of an important oncogenic mutant, the relationships between these events and their functional significance in a setting with established biological relevance. A previous quantitative phosphoproteomics study [37] detected 100 phosphorylation sites on 88 proteins in these two cell lines. In this study, we used microarrays to measure global expression differences between these cells, identifying 1,623 differentially expressed genes (Table S1).

In order to uncover links between the phosphorylation and transcriptional changes, we identified a set of transcriptional regulators that were most likely to be differentially active in the two cell types and responsible for the transcriptional changes. Our approach integrates sequence information with epigenomic and expression differences between the cell lines. We collected DNase-Seq data in each cell line and found 12,807 regions that showed quantitative changes in hypersensitivity. About 68% of these sites were hypersensitive in only one condition, while the rest were hypersensitive to different extents in the two cell lines. We then scanned these differentially hypersensitive regions for matches to known DNA binding motifs to compute affinity scores of the motifs in these regions. To select a subset of these motifs most likely to drive expression differences, we scored each motif using a univariate regression model relating its affinity scores in the differentially hypersensitive regions to differential mRNA expression of genes within 40 kb [24]–[26]. Each regression equation evaluated the function of one protein over potentially hundreds of proximal and distal regulatory elements associated with the set of differentially expressed genes. As a result, we were able to discover trans-acting transcription factors from the changes between two cell types.

**Figure 2:**
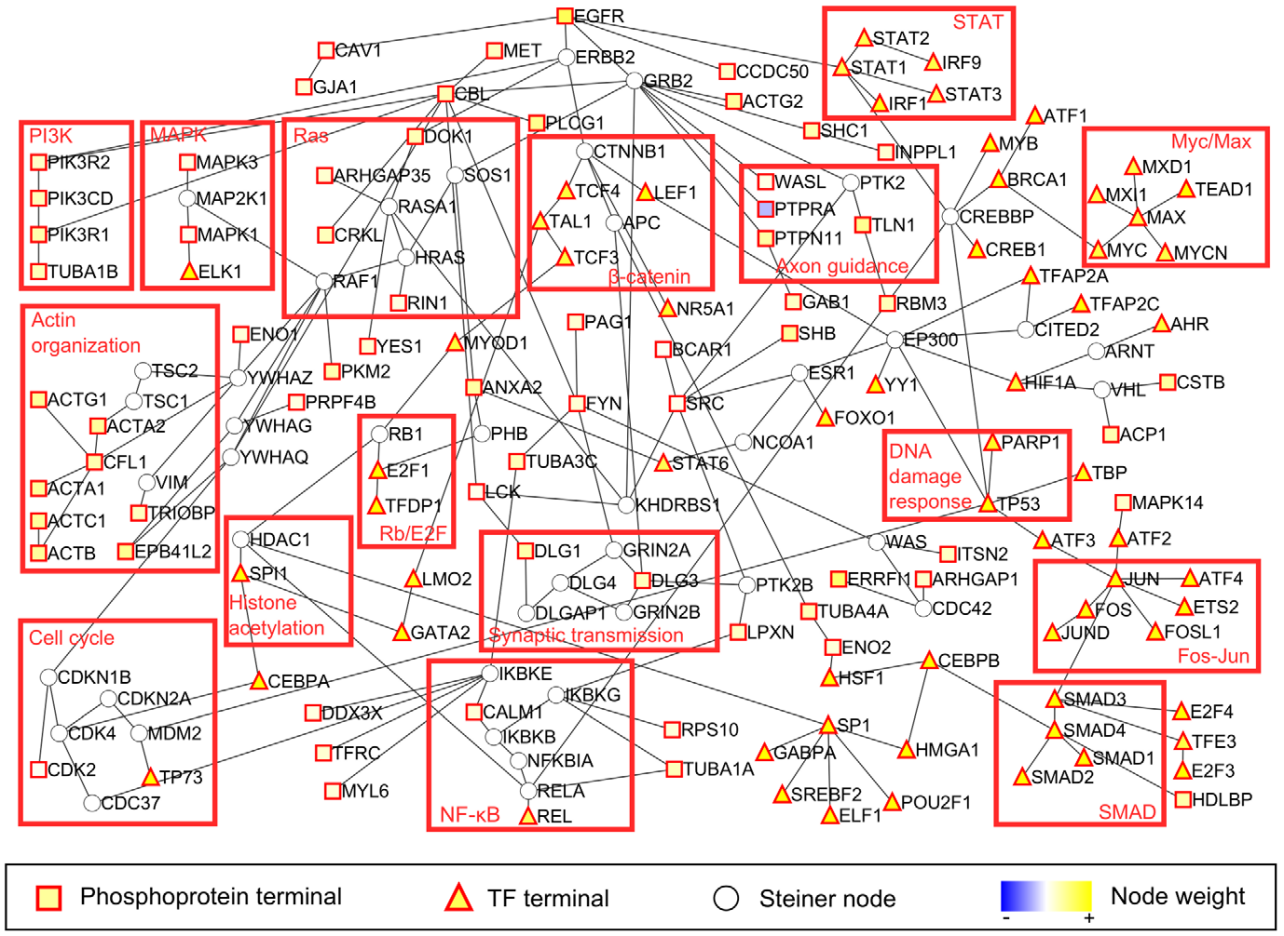
PCST constructed from the U87 datasets. This is a composite network representing the union of the optimal solution to the original PCST problem and 10 suboptimal solutions where 15 percent of the nodes must be different from the optimal solution. TF: transcription factor. Node weight: the log2 fold changes in phosphorylation from the phosphoproteomic data comparing U87H to U87DK cells, or values from the expression regression procedure using the mRNA microarray, DNase-Seq and transcription factor motif data. The absolute value of node weights was used as penalty values for the PCST algorithm.

With this approach, we identified 185 significant motifs that mapped to 297 proteins in the interactome (see Materials and Methods). These proteins represent candidate factors that show either partial or complete changes in their activity between the cell lines. Proteins identified either through phosphoproteomic measurements or by this regression analysis were provided as input to the PCST algorithm along with the interactome data (Figure 1B). The algorithm was run multiple times to find an optimal PCST and a set of ten related sub-optimal solutions. Merging these network solutions, we obtained the network presented in Figure 2. Adopting the PCST terminology, we refer to the phosphoproteins and transcription factor candidates as “termini” and the nodes supported only by the network analysis as “Steiner nodes”.

The interactome we used was derived from iRefIndex [38], a protein interaction database consolidating records from many primary interaction databases such as BIND [39], BioGRID [40], HPRD [41], IntAct [42] and MINT [43]. To account for the fact that the interactions vary in their reliability, we used the Miscore algorithm [44] to assign to each edge in the interactome graph a cost that is inversely related to a likelihood score for the interaction, taking into account the experimental methods that detect the interaction, the type of the interaction, and the number of publications supporting the interaction. The PCST algorithm aims to minimize the sum of the costs of edges required to connect the termini. Therefore, the optimal PCST solution represents a most likely network that links together the protein termini supported by experimental data.

### The PCST solution recapitulates known biology of glioblastoma

The PCST network constructed from the U87 EGFRvIII dataset consists of 199 edges and 172 nodes, of which 65 are phoshoprotein termini and 63 are transcription factor termini (Figure 2). It gives a high-level view of the overall biological processes involved. Such processes include signaling pathways known to be activated downstream of EGFRvIII in U87MG cells, such as the phosphatidylinositol 3-kinase (PI3K) pathway and the Ras-Raf-MEK pathway (HRAS, RAF1 and various mitogen-activated protein kinases (MAPK) in Figure 2) [45], [46]. Others are more general processes induced by EGFRvIII that are known to contribute to tumor development, such as regulation of the cell cycle [45], DNA damage response [36], actin cytoskeleton organization and cell motility [47], [48]. We also note that the network includes the following two cell surface receptors: MET (met proto-oncogene), which contains a tyrosine phosphorylation site, and ERBB2 (v-erb-b2 erythroblastic leukemia viral oncogene homolog 2, neuro/glioblastoma derived oncogene homolog (avian)), for which no phosphorylated tyrosine residue was detected. Both were previously shown to be cross-activated by EGFRvIII [37], [49].

Intriguingly, the network includes two subnetworks containing neurological processes previously linked to GBM: synaptic transmission and axon guidance. The neurotransmitter glutamate, which mediates synaptic transmission through the N-methyl-D-aspartate glutamate receptors 2A and 2B (GRIN2A and GRIN2B) that appear in the PCST network, can promote glioma cell growth [50], and this pro-proliferative effect in U87MG cells is due to EGFR signaling [51]. In addition, genes associated with synaptic transmission and axon guidance are detected as frequently altered in large-scale sequencing and gene expression analysis of human GBM [52]. Although the genetic and transcriptional changes are associated with the neural subtype of GBM, where the EGFRvIII mutation was not found [53], our findings raises the possibility that EGFRvIII-induced post-translational modification may also alter these processes.

Our approach also recovered a number of transcriptional regulators already known to be involved in EGFRvIII signaling. For example, our network identifies the transcription factor signal transducer and activator of transcription 3 (STAT3), which has been previously implicated in EGFRvIII induced transformation [54]. The STAT proteins are unusual in that they were included due to three types of evidence: the expression regression procedure, a tyrosine phosphorylation site on STAT3 (Y705) [37] and direct interactions with EGFR (both wild-type EGFR and EGFRvIII) [54]–[57]. In contrast, the other transcription factors in the PCST network were present solely because of our modeling approach. Several of the prominent transcription factors in the PCST network, such as nuclear factor (NF)-κB (RELA and REL), activator protein 1 (AP-1; consisting of c-Fos (FOS), c-Jun (JUN), ATF proteins), and CCAAT/enhancer binding protein (C/EBP; family member C/EBP-β (CEBPB)), have been reported to be activated in EGFRvIII-expressing glioblastoma cells in an independent study [58]. Furthermore, the oncoprotein MYC, which was captured by our network, is known to be expressed in the U87MG cells and its transcriptional activity contributes to the undifferentiated state and consequently the high tumorigenicity of this cell line [59]. We emphasize that neither the phosphoproteomic data nor the transcription profiling data alone would have suggested roles for these proteins. No phosphorylation sites on these transcription factors were reported by mass spectrometry, and even the tyrosine phosphorylation site on STAT3 (Y705) showed less than 10% change in response to EGFRvIII expression. Nevertheless, in our network solution these transcription factors are prominently featured, demonstrating the value of the PCST algorithm for integrating these data by the interactome. By contrast, standard analysis of the promoter sequences of the differentially expressed genes for enriched transcription factor motifs resulted in only the cell cycle regulator E2F and a zinc finger protein (Table S2).

### The PSCT solution identifies proteins relevant to GBM

**Figure 3:**
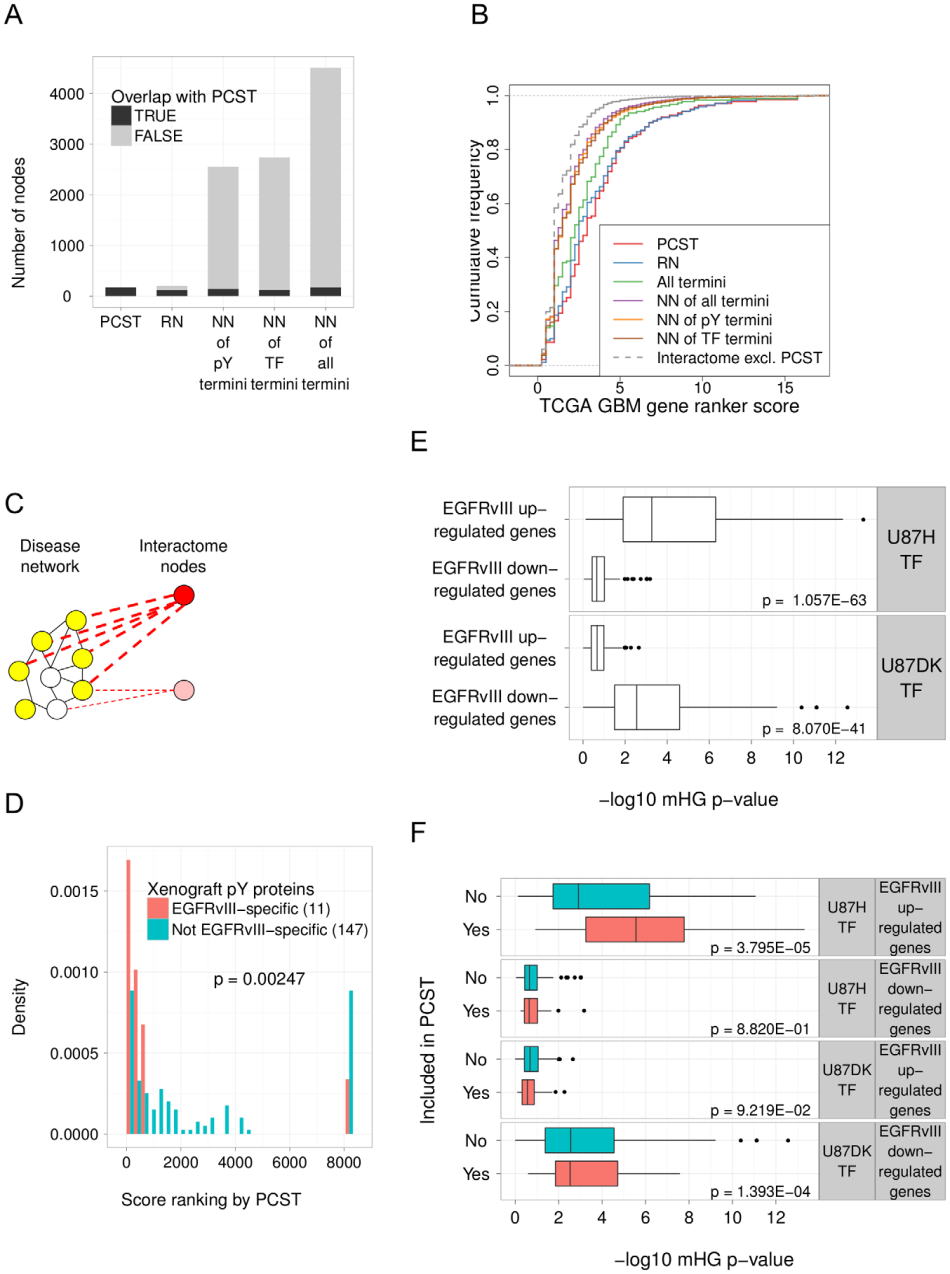
The PCST solution network is compact, relevant to GBM and specific to EGFRvIII. A. The number of nodes in networks constructed from multiple approaches and their overlap with the PCST solution. NN of pY termini: the proteins containing phosphorylated tyrosine residues reported by mass-spectrometry and their direct interactors (nearest neighbors) in the interactome. NN of TF termini: transcription factor candidates selected by the expression regression procedure and their direct interactors in the interactome. NN of all termini: the union of pY termini, TF termini and their direct interactors in the interactome. RN: a network constructed by using a flow based approach ResponseNet [16] to connect the pY termini to the TF termini. B. GBM gene ranker scores for nodes included in the PCST solution were significantly higher than the nodes excluded from the PCST solution (labeled as “Interactome excl. PCST”; p<2.2E-16 by Wilcoxon rank-sum test) and compared favorably to the nearest neighbor networks. Higher GBM scores indicate greater relevance to the disease. C. Scoring proteins by connectivity to the PCST solution representing a disease network. The score of each protein, whether the protein is inside or outside of the original PCST network, is the sum of the scores of all its interactions with the nodes in the PCST. Thus a node in the interactome (deep red) with many high confidence interactions to the nodes in the PCST disease network receives a higher score than a node in the interactome (light red) that has fewer or lower confidence interactions to the nodes in the PCST. D. Proteins with EGFRvIII regulated tyrosine phosphorylation in mouse xenografts (red bars) are more closely connected to the PCST solution than the proteins on which the tyrosine phosphorylation levels do not change significantly (green bars). Each protein in the interactome was scored then ranked by its connectivity to the PCST solution constructed from the U87 cell line data as described in B and in Materials and Methods. P-value was computed by Wilcoxon rank-sum test comparing the ranks of EGFRvIII-specific and not EGFRvIII specific phosphorylated proteins. The number of proteins in each category is indicated in parentheses. E. The targets for transcription factors identified in condition-specific DNaseI hypersensitive regions are enriched for genes differentially expressed in response to EGFRvIII. U87H TF: transcription factors that have motif matches in regions with increased DNaseI hypersensitivity in the U87H cells and within 40 kb of transcription start sites. U87DK TF: transcription factors that have motif matches in the regions with higher DNaseI hypersensitivity in the U87DK cells and within 40 kb of transcription start sites. EGFRvIII up- and down-regulated genes: genes that are up- or down- regulated in the TCGA GBM exon array dataset comparing EGFRvIII positive samples to wild-type EGFR samples. For each TF, we computed a minimum hypergeometric (mHG) p-value that tested for the probability that the set of target genes are differentially expressed in the TCGA GBM samples by chance. Top panel: U87H TF targets are more enriched (smaller mHG values) in EGFRvIII up-regulated genes than in EGFRvIII down-regulated genes. Bottom panel: U87DK TF targets are more enriched in EGFRvIII down-regulated genes than in EGFRvIII up-regulated genes. P-values were computed by Student's t-test comparing the mHG p-values on EGFRvIII up- and down-regulated genes for each set of TF. F. The transcription factors included in the PCST solution are more enriched in EGFRvIII-induced differential gene expression than the transcription factors excluded from the PCST. Each set of U87H TF and U87DK TF were further divided into whether they were included in the PCST solution, denoted by the “Yes” and “No” categories. First panel: targets of U87H TF included in the PCST solution have stronger enrichment in EGFRvIII up-regulated genes than targets of the TF excluded from the solution. Fourth panel: targets of U87DK TF included in the PCST solution have stronger enrichment in EGFRvIII down-regulated genes than targets of the TF excluded from the PCST. Second and third panel: with respect to the comparison between U87H TF targets and EGFRvIII down-regulated genes, or between U87DK TF targets and EGFRvIII up-regulated genes, the TF included in the PCST do not show significantly stronger enrichment than the TF excluded from the PCST. P-values were computed by Student's t-test comparing the mHG scores of TF included in the PCST and TF excluded from the PCST.

The PCST solution network was constructed by finding a small number of proteins that directly or indirectly interact with the set of termini in order to explain the measured changes between U87H and U87DK cells. Compared to networks constructed from proteins that directly interact with the set of phosphorylated proteins, transcription factor candidates or both (“nearest neighbor” networks), the number of nodes in the PCST solution network is about 4% to 7% of these nearest neighbor networks (Figure 3A). To systematically assess whether the highly compact view of the experimental data provided by the PCST solution is able to capture the underlying biological process, we compared the PCST solution to the alternate network approaches with respect to curated collections of genes known to be relevant to GBM. The Cancer Genome Atlas (TCGA) GBM 6000 Gene Ranker [60] scores and ranks over 7,600 genes for relevance to GBM based on gene expression, mutation, pathway analysis and literature curation. Figure 3B illustrates the scoring of all nodes reported by the various network solutions in addition to the PCST. As expected, the set of all termini (those directly detected in the phosphoproteomic data and inferred from differential expression) have high scores. However, we found that the proteins included in the PCST solution scored even higher, and also scored higher than the set of proteins in the input interactome that were excluded from the PCST solution (Figure 3B). A possible trivial explanation for this enrichment could have been that all proteins that interact with the termini are more relevant than the non-interactors, as a result of the biology or biases in the gene ranker curation. To explore this possibility, we compared the PCST to the nearest neighbor networks of various sets of termini and to ResponseNet, a previously published optimization-based network construction algorithm [16]. Both ResponseNet and PCST outperform the nearest neighbor networks. As the PCST solution is smaller than that of ResponseNet and achieves slightly better performance, it is likely to be better for guiding experiments. In summary, the PCST network approach selected a combination of experimentally determined proteins and “hidden” proteins that are relevant to GBM.

### The PCST solution is specific to EGFRvIII at the signaling and transcription level

Since GBM is a heterogeneous disease where the EGFRvIII mutation is among the most common mutation of EGFR [53], we used independent datasets to validate that our network was specific to the EGFRvIII mutation among GBM cases. At the signaling level, we utilized a recently published global phosphoproteomic analysis of mouse GBM xenografts [61]. This study identified 225 tyrosine phosphorylation sites on 168 proteins, and is independent of the U87 cell line data that were the input to the PCST network. Comparison of xenografts expressing high level of EGFRvIII to xenografts expressing normal level of wild-type EGFR resulted in 11 differentially phosphorylated proteins. We asked how closely connected (in the protein interaction network) these EGFRvIII-specific phosphorylation events detected in xenografts were to proteins in the PCST network derived from the U87MG cell lines (Figure 3C). 158 of the 168 proteins in this dataset are present in the set of 11,637 proteins in the interactome that we could score for connectivity to the PCST. We found that 10 out of the 11 (91%) EGFRvIII-specific phosphorylation events fell in the top 6.4% of the proteins closest to the PCST compared to 36.7% of the phosphorylation events that were not EGFRvIII-specific (p<0.003 and Figure 3D). This suggests that the PCST solution, although constructed using experimental data from a tissue culture model, is closely related to protein signaling changes induced by the same oncogenic mutation *in vivo*.

To determine if the transcription factors in the PCST solution contribute to the transcriptional response to EGFRvIII mutation in human patients, we used publicly available TCGA GBM data [53], [62] to compare gene expression alterations in patients with and without the vIII mutation. More specifically, we extracted a set of samples that share mutation status with the U87 cells (p53 wild-type and p16 null) and classified those samples by EGFRvIII status based on EGFR exon probe set expression. Overall, the differences in expression between tumors with wild-type EGFR and those with the vIII mutation were small, which is consistent with the heterogeneous nature of GBM (Figure S2). We tested whether the targets of transcription factors in the PCST solution were associated with vIII-associated changes in gene expression as described below. First, we collected potential targets of each transcription factor in TRANSFAC by scanning for motif matches in genomic regions that show higher DNaseI hypersensitivity in each of the U87H and U87DK cells within 40 kb of transcription start sites. We then ranked all genes by the log-fold change between patients with and without the EGFRvIII mutation and used the minimum hypergeometric statistic (mHG; [63]) to ask whether the ranks of the putative targets of that transcription factors differ from the distribution expected from whole genome background by chance. Thus, for each transcription factor identified in the U87H and U87DK cells we obtained a p-value representing the significance of finding EGFRvIII responsive up- and down-regulated genes in the putative targets of this factor, depicted in Figure 3E.

The EGFRvIII up-regulated genes in the TCGA samples are more strongly enriched (lower mHG p-values) for targets of the transcription factors identified in the U87H cells (U87H TF) than the EGFRvIII down-regulated genes (Figure 3E top panel). Conversely, for the set of transcription factors that have motif matches in regions with increased DNaseI hypersensitivity in the U87DK cells (U87DK TF), their targets are more strongly enriched for EGFRvIII down-regulated genes than the up-regulated genes (Figure 3E bottom panel). These results support the utility of differential hypersensitivity for identifying transcription factors relevant to human tumors even though the DNase-Seq data were generated exclusively from cell lines and the EGFRvIII mutation has a modest effect on the global transcriptional programs in patients.

To assess the role of the PCST in selecting biologically relevant transcription factors, we classified factors based on whether they were included in the PCST solution. The U87H transcription factors included in the PCST network solution have even stronger enrichment in the EGFRvIII up-regulated genes than those factors excluded from the PCST solution (Figure 3F first panel), and the U87DK transcription factors included in the PCST show stronger enrichment in genes down-regulated in EGFRvIII patients than the excluded factors (Figure 3F fourth panel). Such differences between the included and excluded transcription factors are not observed for the associations between the U87H transcription factor targets and the EGFRvIII down-regulated genes, or between the U87DK transcription factor targets and the EGFRvIII up-regulated genes (Figure 3F second and third panel). This finding suggests that for transcription factor candidates selected based on motif evidence in open chromatin and correlation with target expression in cell line models, those that can be linked to upstream signaling changes are more likely to affect expression changes *in vivo*.

### Network connectivity analysis can be used to prioritize experiments

Having established that our approach recovered prior knowledge related to GBM, we used the network to prioritize experiments for testing new points of intervention in the network. We selected protein targets based on two criteria: (1) a quantitative score that measures how closely connected they were to the nodes in the PCST in the interactome (Figure 3C) and (2) whether they were targets of commercially available small molecules [64]–[66]. Our final list included fifteen nodes, all of which were among the 30% of the interactome most closely connected to the PCST. Eight of these were extremely high-scoring nodes (of which seven are in the original PCST and one is closely connected to the PCST), one was an intermediate scoring nodes, and six were lower-scoring nodes (Table 1). We treated the U87DK and U87H cells with small molecule antagonists for these highest-, intermediate- and lower-scoring nodes (“high-ranked”, “mid-ranked” and “lower-ranked” targets) at a wide range of concentrations and measured the resulting cell viabilities relative to those of vehicle treatment.

**Table 1:** High-, mid- lower-ranked nodes by the PCST network and the experiments used to validate their importance.

Experiment	Small molecule inhibitor	Antibody	Target	Target rank	Target type
Viability	Dasatinib		SRC	3	High-ranked target
			FYN	12	
ChIP-Seq		sc-585x	EP300	4	High-ranked target
Viability	ICG-001		CREBBP	5	High-ranked target
Viability	4-hydroxytamoxifen (4-OHT)		ESR1	15	High-ranked target
Viability	suberoylanilide hydroxamic acid (SAHA)		HDAC1	19	High-ranked target
Viability	PKF118–310		CTNNB1	21	High-ranked target
Viability	ammonium pyrrolidinedithiocarbamate (PDTC)		NFKB1	23	High-ranked target
Viability	17-N-Allylamino-17-demethoxygeldanamycin (17-AAG)		HSP90AA1	26	High-ranked target
Viability	SB-505124		TGFBR1	193	Mid-ranked target
Viability	SB-431542		TGFBR1	193	Mid-ranked target
			ACVR1B	1695	
Viability	Rapamycin		MTOR	698	Lower-ranked target
Viability	D4476		CSNK1A1	875	Lower-ranked target
Viability	Harmine		DYRK1A	2232	Lower-ranked target
			MAOA	8508.5	
Viability	Paclitaxel		TUBB1	3582	Lower-ranked target

For cell viability assays, the inhibitors used are listed. Note that some inhibitors have multiple targets. For ChIP-Seq, the antibody used is listed.

**Figure 4:**
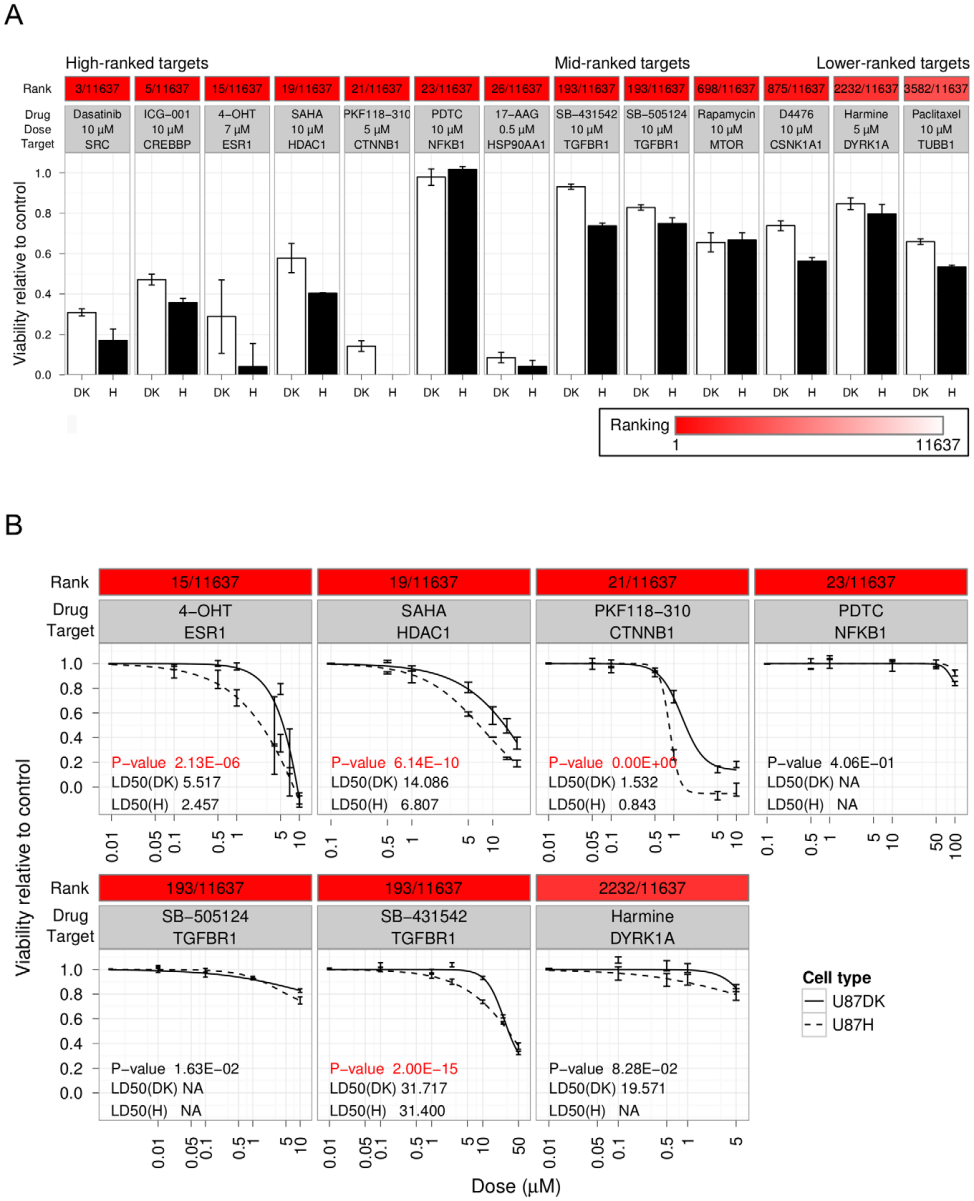
Validation of targets predicted by network connectivity by cell viability assays. A. Cell viability for treatment with compounds targeting high-scoring nodes (high-ranked targets), intermediate-scoring nodes (mid-ranked targets) and low-scoring nodes (lower-ranked targets), at 0.5 µM concentration of 17-AAG, 5 µM for harmine (due to low solubility in DMSO) and 10 µM concentration of others. The color bar at the top of each target corresponds to its relative ranking within the interactome. B. Dose response curves of compounds targeting high-scoring nodes and lower-scoring nodes for those that can be fitted to the four-parameter log-logistic model (lack-of-fit test p-value>0.05). P-values between cell lines were computed by comparing the model where one curve was fitted to the data from each cell line to the null model where one shared curve was fitted to the data from both cell lines.

We first compared the effects of all the compounds at a concentration of 10 µM (except for harmine, which was only soluble at 5 µM in the organic solvent DMSO) (Figure 4A). With the exception of PDTC, which targets NF-κB (NFKB1), all compounds targeting the highest-ranked nodes reduced viability by at least 40% at 10 µM. In the cases of 4-OHT (targeting the estrogen receptor ESR1), 17-AAG (targeting heat shock protein 90kDa α HSP90AA1) and PKF118–310 (targeting β-catenin CTNNB1), more than 70% of the cells were killed at concentrations lower than 10 µM. The effects on the mid- and lower-ranked nodes were more modest, resulting in 10 to 50% reduction of viability. One trivial explanation for such difference would be if the compounds for the intermediate and lower-ranked targets happen to require higher doses in all biological contexts. However, this was not likely the case because these concentrations, which were only weakly effective in the U87-derived cells, were a few orders of magnitude *higher* than the GI50 values (the concentration that inhibit growth by 50%) in the NCI60 panel of cancer lines as reported by the In Vitro Cell Line Screening Project (IVCLSP) of the National Cancer Institute Developmental Therapeutics Program ([67] and http://dtp.nci.nih.gov/). By contrast, the doses we used to target the highest-ranked nodes were within the range of GI50 values (Figure S3). Therefore, we conclude that the highest-ranked targets have stronger effects on the viability of the U87 cells than the targets with lower ranks.

To further understand the behavior of these compounds on the U87 cells, we fit the measured dose responses to a four-parameter log-logistic (4-PL) function. For compounds that could be fit to the 4-PL model (lack-of-fit test p>0.05), we compared parameters of the fitted curves between U87H and U87DK cells (Figure 4B). Compounds for three out of the four highest-ranked targets, SAHA (targeting histone deacetylase HDAC1), 4-OHT (targeting estrogen receptor ESR1) and PFK118–310 (targeting β-catenin CTNNB1) were more toxic under EGFRvIII expressing condition (p<0.01), with LD50 values an average of two-fold higher in the DK cells. In contrast, cells treated with two of the three compounds for the lower-ranked targets, SB-505124 (targeting TGF-β receptor 1 TGFBR1, ranked 193 out of 11,637) and harmine (targeting dual-specificity tyrosine phosphorylation-regulated kinase DYRK1A, ranked 2,232 out of 11,637), exerted similar effects on the two cell lines. SB-431542 (for the lower-ranked targets TGFBR1, ranked 193 out of 11,637, and activin receptor type-1B ACVR1B, ranked 1,695 out of 11,637) appeared to exert a significantly different effect in the presence of EGFRvIII, but the difference in LD50 values was only 1% and more than 30 µM was required to reduce viability by 50% compared with much lower doses for the high-ranked targets. These results demonstrate that the compounds targeting the high-ranked nodes are more likely to have strong effects on cell viability and be associated with large differential sensitivity between two cell lines, implying the high-ranked targets give rise to important signaling differences induced by EGFRvIII.

### ChIP validated the relevance of transcriptional coregulator p300

Since transcriptional regulation is the first step that defines the long-term behavior of the cell including tumorigenesis, we sought to identify the transcriptional regulators responding to oncogenic signaling. In addition to several transcription factors known to be induced by EGFRvIII, our PCST network also included novel putative transcriptional regulators. Using the same scoring procedure as for ranking targets for small molecules (Figure 3C), we selected the highly ranked transcriptional co-regulator p300 (EP300) for experimental validation (Table 1). p300 is a particularly interesting candidate because it is the highest ranked transcriptional regulator (out of 937 annotated transcription factors, co-activators and co-repressors ranked by the network), and although it appears in the network it is not a terminal, i.e., it was not identified as a transcription factor candidate, nor does it contain phosphorylated tyrosine residues. We chose to perform a chromatin immunoprecipitation sequencing (ChIP-Seq) experiment for p300 since determining the genome-wide binding locations of a transcriptional regulator may suggest its biological functions and shed light on its regulatory mechanism. Both aspects could validate the predicted relevance of p300 in our experimental system and therefore demonstrate the capability of our method to uncover important regulators not present in the original signaling and transcription data.

We therefore performed a ChIP-Seq experiment in the U87H cells to identify the genes targeted by p300. 60.8 million uniquely mapped reads of 36 bp were obtained (Table S3). Peak calling by MACS reported 28,721 peaks at low stringency (p-value threshold 1E-05) and 7,657 peaks at high stringency (p-value threshold 1E-07), mapped to 6,391 and 1,969 genes within a 10 kb window, respectively.

**Figure 5:**
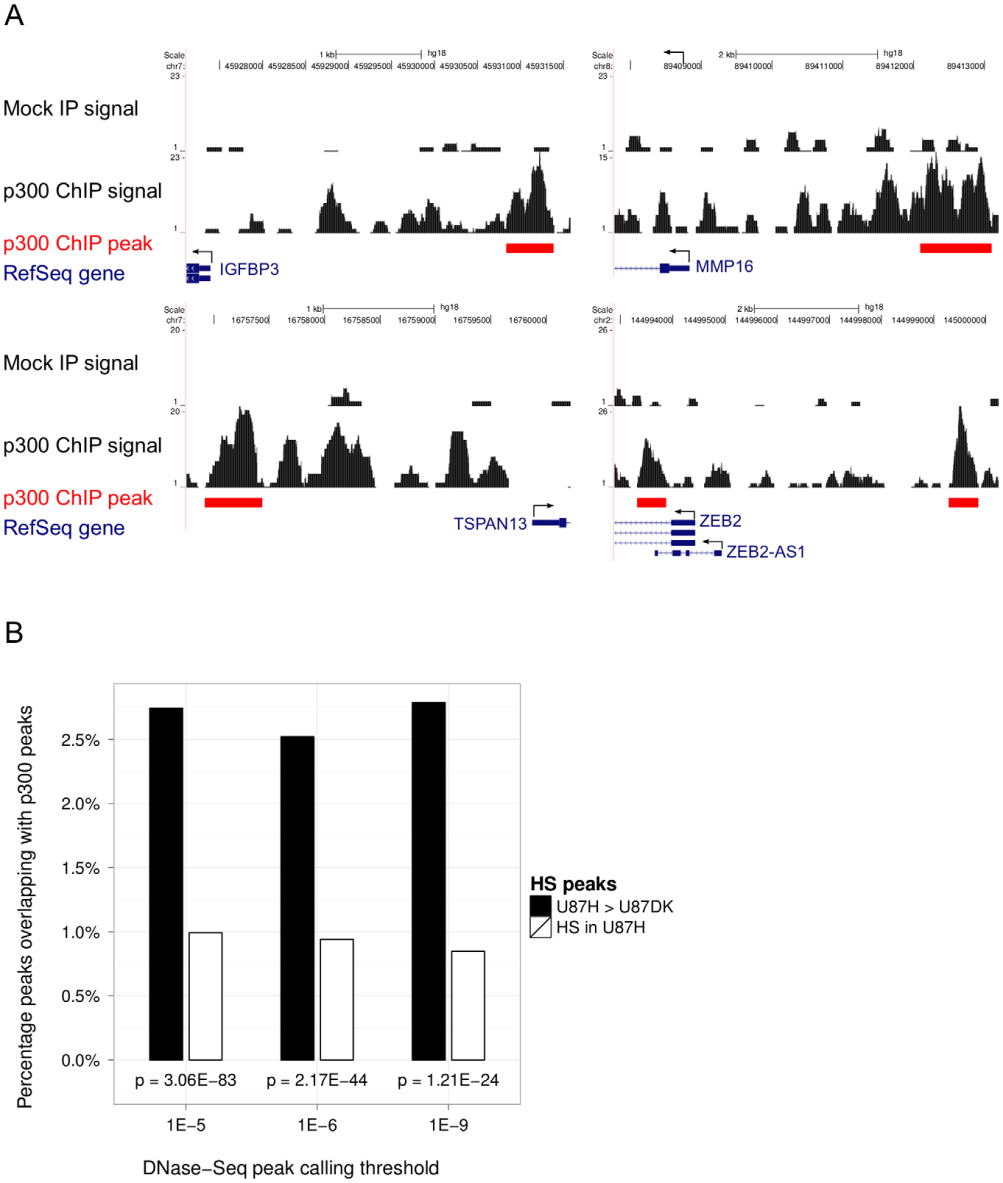
ChIP-Seq reveals functional role of p300. A. EMT marker genes bound by p300 in U87H cells. Shown are genome browser tracks for p300 bound regions near several EMT marker genes, where the horizontal axis represent coordinates along the genome and the height of the solid area represents the number of ChIP-Seq reads mapped to a position in the genome. For each region we show this signal from the ChIP sample that used an antibody specific to p300 (bottom track) and the signal from the sample that used an IgG antibody for non-specific binding (top track). Arrow indicates direction of transcription. B. Regions that are more hypersensitive (HS) in the U87H cells were significantly enriched for overlap with p300 binding regions (p<1E-05) compared to a background of all regions called hypersensitive in U87H cells, for a range of peak calling thresholds of hypersensitivity specified on the x-axis tick marks. Enrichment p-values computed by Fisher exact test are indicated immediately below each set of bars.

p300 does not directly bind DNA but is instead recruited to its targets by sequence-specific DNA-binding proteins. Therefore, the PCST algorithm selected it solely by virtue of its protein-protein interactions. Nevertheless, if the network is correct, we should be able to detect the consequences of its recruitment to the DNA. Indeed, we found evidence that p300 is actively involved in chromatin remodeling of these cells: p300 binding sites are significantly enriched in regions that increase in hypersensitivity in the U87H cells compared to U87DK cells (p<1E-23; Figure 5B), suggesting that p300 may regulate transcriptional changes between the two cell types.

Our networks also included the DNA-binding proteins that recruit p300 to its targets. To test these predictions, we examined the sequence motifs present in the highest confidence p300 targets, defined as those regions that were present in the ChIP-Seq data and showed a change in DNaseI hypersensitivity between the cell lines. We used cross-validation to rank motifs by their ability to predict which differentially hypersensitive sequences were p300 targets (see Materials and Methods). Of the 151 non-redundant vertebrate TRANSFAC motifs, 52 correspond to proteins known to interact with p300; 33 of these were inside the PCST and 19 were outside of it. Among the 20 motifs that were the most predictive of p300 recruitment, at least 8 correspond to proteins inside the PCST (enrichment p-value = 0.039 by Fisher exact test), while only one was outside of the PCST (enrichment p-value = 0.94 by Fisher exact test) (Table S4). These motif results, together with the observed association between p300 binding and hypersensitive sites, provide solid support for the role assigned to p300 in the network as a transcriptional regulator responding to EGFRvIII.

Gene Ontology enrichment analysis of the p300 targets identified by ChIP-Seq revealed the potentially important role of this protein in regulating the transcriptional consequences of the EGFRvIII mutation (Table 2), specifically in cellular adhesion and response to hormone. Neither of these categories is enriched in p300-bound sites in the two other cell types in ENCODE [19] for which ChIP-Seq data were available (Table S5), suggesting that the functional role of p300 is likely to be specific to our system.

**Table 2:** Enriched Gene Ontology (GO) categories of p300 target genes in U87H cells.

GO Term	Description	P-value	% FDR	Official gene symbol
GO:0007155	cell adhesion	1.15E-03	2.05	AEBP1, NRP1, THRA, MYBPC3, CUZD1, CDH22, WISP1, ROBO1, DGCR6, CNTNAP2, ZYX, LOXL2, DLG1, SPON1, ROCK1, PTPRF, NRXN2, TRPM7, PCDHB1, SDK1, ACTN1, PTPRT, NRXN1, PSEN1, HAS1, GPR56, VCAN, SEMA4D, CD226, PARVA, PKHD1, ITGAE, TNC, ITGA11, ITGB2, PKD1L1, ITGAM, CLDN14, CDH5, ITGBL1, LY6D, SORBS1, PTK2B, COL27A1, TTYH1, ITGB6, BAI1, COL6A2, TSTA3, THBS2, TECTA, COL18A1, MAG, FLRT1, COL5A3, RAPH1, CLDN23, LYVE1, COL14A1, LAMA3, CDH16, ITGA6, ERBB2IP, CD300A, DSG3, PKP4, FCGBP, MUC5AC, CDH11, MUC16
GO:0022610	biological adhesion	1.16E-03	2.07	AEBP1, NRP1, THRA, MYBPC3, CUZD1, CDH22, WISP1, ROBO1, DGCR6, CNTNAP2, ZYX, LOXL2, DLG1, SPON1, ROCK1, PTPRF, NRXN2, TRPM7, PCDHB1, SDK1, ACTN1, PTPRT, NRXN1, PSEN1, HAS1, GPR56, VCAN, SEMA4D, CD226, PARVA, PKHD1, ITGAE, TNC, ITGA11, ITGB2, PKD1L1, ITGAM, CLDN14, CDH5, ITGBL1, LY6D, SORBS1, PTK2B, COL27A1, TTYH1, ITGB6, BAI1, COL6A2, TSTA3, THBS2, TECTA, COL18A1, MAG, FLRT1, COL5A3, RAPH1, CLDN23, LYVE1, COL14A1, LAMA3, CDH16, ITGA6, ERBB2IP, CD300A, DSG3, PKP4, FCGBP, MUC5AC, CDH11, MUC16
GO:0032870	cellular response to hormone stimulus	1.65E-03	2.94	ADCY3, IRS2, WDTC1, ADCY2, THRA, ADCY5, PRKCI, AP3S1, IGF2, CUZD1, TRH, IRS1, GNG8, GRB10, SORBS1, GNB1, PRKAR1B, GHRL, GNAS, HDAC9
GO:0009755	hormone-mediated signaling	2.34E-03	4.16	GNG8, ADCY3, ADCY2, THRA, GNB1, ADCY5, PRKAR1B, GHRL, GNAS, CUZD1, TRH

1,969 genes within 10 kb of 7,657 high stringency peaks called by MACS (p<1E-07) [112] were input into the DAVID functional annotation tool [120] to identify enriched Biological Process terms with FDR <5%.

## Discussion

### Linking signaling and transcription data by molecular interactions generates novel mechanistic hypotheses

We have shown that the PCST solution provided an integrated view of the biological processes in the EGFRvIII network leading to directly testable predictions. Our analysis revealed proteins whose activities we subsequently targeted with small-molecule inhibitors to block the growth of tumor cell lines. In addition, the PCST solution network identified transcriptional regulators enriched at hypersensitive sites. Many of the proteins we targeted did not appear in the phosphoproteomic and transcriptional profiling data but were selected among thousands of other proteins in the interactome graph that interact directly or indirectly with the hits from the experiments. In particular, a network of direct interactors of the phosphorylated proteins contains 2,554 nodes. In the absence of a network optimization approach, it would be extremely difficult to prioritize experiments. Using the PCST approach, we were able to integrate the phosphoproteomic data with additional transcriptional data and provide a ranked list of proteins for experiments. Targets far from nodes in the PCST were less likely to exert differential cytotoxic effects in response to the oncogenic mutation, and in the cases in which targeting these nodes were cytotoxic, their effects tended to be weaker. Therefore, our approach provides a powerful way to prioritize targets based on experimental datasets that represent different aspects of the cell state such as protein signaling, chromatin conformation, and transcription output.

Our network-based approach was also able to identify transcriptional regulators that could not be found by other methods. Standard promoter analysis of the differential expressed genes in the current datasets yielded little information about potential DNA binding proteins besides the cell cycle regulator E2F and a zinc finger protein (Table S2), whereas integrating data from upstream signaling allowed us to identify promising candidates. The experimental validation of p300 exemplifies the power of our approach. p300 was not a hit in the tyrosine phosphoproteomic dataset, nor was it a sequence-specific DNA binding protein for which a motif can be correlated to differential mRNA expression from the regression analysis. However, it was included in the network due to its connectivity to the measured signaling events and to the sequence specific transcription factors. Identifying genome-wide binding locations of p300 by ChIP-Seq provided experimental support for its role in chromatin remodeling and tumorigenic processes.

### Functional roles of p300

At the genome-wide level, p300 targets were found to be enriched in genes involved in the response to hormone. Little is known about the role of p300 in regulating hormone response genes transcriptionally. However, it is known that p300 associates with multiple nuclear hormone receptor proteins and functions as a co-regulator [68], [69]. Our observation that nuclear receptor genes are p300 targets may represent a mechanism for the autocrine loop observed in EGFRvIII expressing glioma cells [70]. p300 targets were also associated with the process of cellular adhesion. In particular, several p300 target genes that were differentially expressed in the presence of EGFRvIII (Figure 5A) are well-characterized markers for the epithelial-mesenchymal transformation (EMT), a process that is known to alter cellular adhesion [71]. We observed that in the presence of EGFRvIII the cells have poor attachment to the tissue culture plate, consistent with alteration in cellular adhesion and possibly a partial mesenchymal phenotype. Therefore, our data point to the potentially important role of p300 in transcriptional regulation in our system and suggest a mechanism for the mesenchymal properties displayed by GBM cells [72], demonstrating how following up on high-ranked transcriptional regulators by ChIP-Seq can lead to new biological hypotheses.

The sequence specific factors responsible for p300 recruitment to EMT-related genes remain to be found. C/EBP-β (CEBPB) and STAT3 have previously been shown to synergistically induce mesenchymal transformation of glioma cells [73]. However, in our data, the levels of C/EBP-β transcript did not change in response to EGFRvIII expression and the phosphoproteomic data showed no significant change in the levels of activated STAT3. Our network results suggest a possible explanation for these findings. The network includes an interaction between C/EBP-β and SMAD4, and SMAD4 is known to repress the transactivation function of C/EBP-β [74]. We also note that the SMAD4 mRNA level is reduced by five-fold in the presence of EGFRvIII. Together, these data suggest that EGFRvIII expression leads to a decrease in SMAD4. This in turn activates C/EBP-β, which recruits p300 to EMT genes.

### Therapeutic and mechanistic insights from effective high-ranked targets

We tested the effects of seven compounds targeting high-ranked nodes predicted by the PCST solution, and six of these resulted in significant reduction in cell viability. These compounds represent both known and novel therapeutic agents for GBM (Table 3). Of these agents, dasatinib has the best-characterized effect on EGFRvIII glioblastoma. Dasatinib has anti-tumor effects on EGFRvIII-expressing glioblastoma models, including inhibition of invasion and induction of apoptosis [34] although its anti-proliferative effect has also been reported in U87 cells expressing wild-type EGFR [75]. Currently several clinical trials are ongoing for mono- and combination-therapy of dasatinib in GBM [76]. We used dasatinib to target the SRC and FYN kinases, and these have previously been reported to be activated by EGFRvIII [34]. The HSP90 inhibitor 17-AAG, which we found to be highly effective at sub-micromolar concentrations, has also been shown to be effective in a variety of human glioma cell lines and glioma models [77]. 17-AAG has entered clinical trials for several cancer types [78], but has not yet been tested in GBM. The HDAC inhibitor SAHA effectively inhibits tumor cell growth in multiple glioma cell lines and mouse models [79], [80], and a phase 2 clinical trial in patients with recurrent GBM showed modest single-agent activity [81]. In addition, co-delivery of SAHA and siRNA against EGFRvIII synergistically induced apoptosis in GBM cells [82]. Our data is the first demonstration that SAHA alone has additional potency in EGFRvIII-expressing cells. At a mechanistic level, this observation may be related to our discovery that the histone acetylase p300 is involved in chromatin remodeling induced by EGFRvIII.

**Table 3:** Summary of anti-tumor therapies corresponding to the high-ranked targets and compounds that are found to exert significant killing, with emphasis on relevance to GBM.

Compound	Target	Current status
Dasatinib	SRC, FYN	Ongoing phase 1 and 2 trials of mono- and combination-therapy for primary and recurrent GBM [76].
ICG-001	CREBBP	Related compound PRI-724 in phase 1 trial of advanced colorectal cancer and pancreatic cancer (trial identifier NCT01302405); no pre-clinical data for GBM.
17-AAG	HSP90AA1	Phase 1, 2, and 3 trials in multiple cancer types but not including GBM [78]; showed efficacy in glioma models [77].
4-OHT	ESR1	A subgroup of GBM patients responded to high dose tamoxifen [84]; multiple phase 2 trials have been completed as part of combination-therapies for GBM [121].
SAHA	HDAC1	Modest single agent activity in a phase 2 trial for GBM [81]; multiple ongoing phase 1 and 2 trials for combination therapy.
PKF118–310	CTNNB1	Showed efficacy in models of multiple cancer types but not yet tested for GBM.

Our experiments also illustrated that the selective estrogen receptor modulator tamoxifen reduced cell viability in both cell types but more potently in the EGFRvIII-expressing cells. Epidemiologic data on the effect of steroid hormone in the etiology of glioma are ambiguous (reviewed in [83]): prior to menopause, women are at lower risk of glioma than men, suggesting a protective role of estrogens; however, the relative reduction in glioma risk in women from the use of exogenous hormone is small and inconsistent. A high dose of tamoxifen was reported to reduce tumor volume and stabilize tumor progression in a subgroup of recurrent malignant glioma patients [84]. At the molecular level, there are considerable discrepancies regarding the expression of ESR1 in human glioblastoma (see recent summary in [83]). Further study is necessary to determine if particular selective estrogen receptor modulators given alone or in combination with other therapies might be more effective than the estrogen receptor modulator alone. Although ESR1 is a well-characterized transcription factor, the algorithm selected it only because of its interactions with other proteins, not because of the presence of the estrogen responsive element (ERE) sequence motif on DNA. Such ERE-independent actions have precedents; ESR1 is known to cross-talk with protein kinase cascades such as those of ERK (extracellular-signal-regulated kinases) MAPK and PI3K [85], [86] and it regulates transcription by interaction with other transcription factors and co-activators [87], [88]. Interestingly, non-genomic signaling by 17β-Estradiol can both stimulate and inhibit apoptosis [89].

In addition to these previously reported compounds, we identified ICG-001 and PKF118–310 – two agents that are effective against the U87 cells and that had not previously been reported in the context of GBM. ICG-001 inhibits CREBBP and was discovered from screening in a colon cancer cell line [90]. A more potent structural relative of ICG-001, PRI-724, entered phase 1 clinical trial for advanced colorectal and pancreatic cancer in February 2011. The 50% growth inhibitory concentration is in the micromolar range for colon carcinoma cells but ten-fold higher in normal colonic epithelial cells (4.43, 5.95, and 70.90 µM on SW480, HCT116 and CCD-841Co cells, respectively [90]), suggesting the greater than 50% reduction of cell viability we observed at 10 µM concentration is likely to be relevant physiologically. PKF118–310 is a potent inhibitor of the interaction between TCF4 (transcription factor 4) and β-catenin (CTNNB1) [91] and has shown growth inhibitory effects in cell line models of prostate cancer [92], osteosarcoma [93], hepatocellular carcinoma [94], and a mouse model of breast cancer [95], but effects on GBM have not been reported. However, there is evidence that the target of this compound is relevant to GBM: Wnt/β-catenin activation positively correlates with the progression of glioma [96], [97] and down-regulation of β-catenin inhibits glioma cell growth [97], [98]. In our system, expression of EGFRvIII leads to two-fold higher sensitivity to PKF118–310, bringing it to the sub-micromolar range of 50% growth inhibitory concentrations reported in osteosarcoma [93] and hepatocellular carcinoma cells [94]. It is therefore of great interest to determine whether a link exists between EGFRvIII status and β-catenin activation state in GBM patients.

### Future perspectives

We have demonstrated a method for uncovering a physical network of proteins and genes that respond to expression of an oncogenic mutation, which we have used to reveal new methods for specifically blocking growth of the tumor cells. Our approach for reconstructing mammalian signaling pathways uses epigenomic data to create a critical link between phosphoproteomic changes and downstream transcriptional events via physical associations from the interactome. Based on these networks, we were able to identify key transcriptional regulators and discover a number of compounds that killed EGFRvIII cells more effectively than control cells.

While our approach has a low false-positive rate, it is possible that it will miss many potential targets that lie far from the PCST. In fact, we found that an inhibitor for one of the lower-ranked targets (TUBB1, ranked 3,582 out of 11,637) did show a modest difference in cytotoxicity between DK and H cells. Such false-negatives arise because they are part of biological responses that are not connected to the available data, either because the interactome is incomplete or because these targets are functioning in biological processes that have little in common with the proteomic data on which we based our study.

The proteomic data selectively identified phosphorylated tyrosine residues, which are relatively rare compared to phosphorylated serine and threonine [99] but display faster and bigger fold changes [99]. However, the PCST formulation can be readily extended to incoporate phosphorylation data on these other residues, other post-translation modification on proteins, as well as other experimentally- and computationally-derived protein activities that can be used as constraints. In a similar vein, we can supplement or even completely replace the physical interactome on which to solve for the PCST by associations derived from purely data-driven approaches, which may create thousands of connections with different strengths of association. The optimization procedure provided by PCST builds parsimonius networks consisting of the strongest associations that satisfy the experimental constraints, providing a sound basis for designing experiments.

Our models, which treat transcriptional changes as downstream of proteomic changes, focused on identifying the differences between two cell types at steady state. In dynamic systems, it will be important to include feedback as the transcriptional changes also lead to changes at the proteomic level. Although differential equations provide a natural way to describe such feedback, these approaches are limited to relatively small systems. For example, a comprehensive differential-equations-based transcriptional and translational network for *Escherichia coli* [100] has been developed, but a genome-wide model for mammalian proteomics and transcription data is not yet feasible. We propose that our approach can be applied to “-omics” data to reduce the complexity of mammalian signaling and transcription to a point where differential-equations-based modeling becomes feasible. The combination of comprehensive “-omics” based analysis followed by quantitative modeling would provide a method for producing highly quantitative predictions of new therapeutic strategies even for a broad range of diseases.

## Materials and Methods

### Cell culture

The human glioblastoma cell lines U87MG expressing high levels of EGFRvIII (U87H, 2 million EGFRvIII per cell) and a kinase dead mutant of EGFRvIII (U87DK, 2 million kinase dead receptors per cell) were generous gifts from Dr. Paul Huang and Dr. Forest White at MIT. Cells were cultured in complete media (Dulbecco's Modified Eagle Medium (DMEM; Mediatech) supplemented with 10% fetal bovine serum, 100 units/mL penicillin, 100 mg/mL streptomycin (Invitrogen), 4 mM L-glutamine) and in a 95% air/5% CO_2_ humidified atmosphere at 37°C. Expression of EGFRvIII and DK receptors were selected by 400 mg/mL G418 (Calbiochem). To enhance cell attachment, tissue culture vessels with the Corning CellBIND surface (Corning) were used.

### Transcription profiling

Total RNA was prepared from the U87MG derived cell lines by the RNeasy Plus Mini Kit (Qiagen) and quantified on the Affymetrix Human Genome U133 Plus 2.0 arrays. Labeling, hybridization, washing and staining were performed following the standard Affymetrix GeneChip protocol. The arrays were hybridized in an Affymetrix GeneChip Hybridization Oven 640 at 45°C at 60 rpm for 16 hours, washed and stained in Affymetrix Fluidics Station 450, and scanned with Affymetrix GeneChip Scanner 3000 7G. Two biological replicates were done for each cell line. The intensity values were normalized using the GC Robust Multi-array Average (gcrma) package [101] in the R BioConductor library and differential gene expression was calculated by the Linear Models for Microarray Data method [102] implemented as the limma package [103] in BioConductor.

### DNase-Seq

The U87DK and U87H cells were seeded in parental media (complete media without G418). After 24 hours, the cells were washed gently with phosphate buffered saline (PBS) and cultured in serum free media for 24 hours. Nuclei extraction and DNaseI digestion followed published protocol [20], [104] for 50 million nuclei for each of the two biological replicates of each cell line. Sequencing libraries were prepared with the Illumina sample preparation kit and 100 to 300 bp fragments were specifically selected by gel electrophoresis. Each biological replicate was sequenced in one lane on a Genome Analyzer II sequencer (Illumina). The 35 bp-long sequencing reads were aligned to the hg18 genome by Illumina's Eland extended software with maximum two mismatches in the first 25 bp. The sequencing and alignment statistics are listed in Table S3.

### ChIP-Seq

The U87H cells were seeded in media without G418. After 24 hours, the cells were washed gently with PBS and cultured in serum free media for 24 hours. Crosslinking and cell lysis were done as previously described [105], and sonication was performed on a Bioruptor NextGen sonication system (Diagenode) with 10 cycles of 30 sec on, 30 sec off at high power setting. p300 ChIP was done on the SX-8G IPStar Automated System (Diagenode) with buffers from the Auto Transcription ChIP kit (Diagenode) following instruction manual version V1_07-10-10. The pre-set IP protocol “ChIP 22 hr IPure16 200vol” was used with 5 hours of antibody coating and 16 hours of ChIP reaction at 4°C. 3 µg of the p300 antibody sc-585x Lot#E2610 (Santa Cruz) was used on 25 µL of the sonicated chromatin diluted with 75 µL of ChIP Buffer T. The ChIP products were reverse-crosslinked at 65°C for 6 hours with occasional vortexing. ChIP DNA was purified by reagents in the Auto IPure kit (Diagenode) but done manually following the IPure kit (Diagenode) instruction manual version V2_12-05-10. Sequencing library was prepared from the purified DNA by the SPRI-te Nucleic Acid extractor (Beckman Coulter) with SPRIworks Fragment Library System I cartridges according to manufacturer's protocol. Enrichment was done with 2× Phusion Master Mix, PE PCR primer 1.0 (Illumina) and a barcoded paired-end PCR primer 2.0. The library was sequenced in one paired-end lane on Illumina Genome Analyzer II. The sequencing reads of 36 bp were aligned to the hg18 genome by the short reads aligner bowtie [106] version 0.12.5 suppressing all alignments for reads that align to more than one location (-m 1). The sequencing and alignment statistics are listed in Table S3.

### Cell viability assay

4,000 cells in 100 µL of parental media were seeded per well in a 96-well CellBIND clear plate. Twenty-four hours later, the medium was aspirated, each well was washed with 150 µL of PBS, and 100 µL of fresh serum-free media (DMEM with no phenol red) containing the indicated concentrations of drugs was added. Six to eight within-day biological replicates were performed for at least three between-day biological replicates for each treatment of each cell line. To make stock solutions of small molecule drugs, dasatinib, rapamycin, paclitaxel (LC Labs), ICG-001, SAHA, SB-431542 (Selleck Chemicals), 17-AAG (AG Scientific), PKF118–310, SB-505124 (Sigma), D4476, and harmine (Cayman Chemical) were dissolved in dimethyl sulfoxide (DMSO), 4-OHT (Sigma) in pure ethanol and PDTC (Sigma) in PBS. All stock solutions were stored in the dark at −20°C and diluted to the desired concentration in cell culture media immediately prior to treatment. After 72 hours of drug treatment, cell viability was measured by the WST-1 reagent (Roche Applied Science). 10 µL of WST-1 was added to each well, the plates were incubated at 37°C for four hours and absorbance at 450 nm was measured by Varioskan Flash Multimode Reader (Thermo Scientific). Raw signals were normalized by a linear mixed-effects model (see below) to eliminate between-day batch effects. Relative viability values were computed as the ratios between the normalized signals of drug-treated cells and the corresponding vehicle control cells. Curve fitting to the four-parameter log-logistic model and statistical tests of fitted parameter values were performed using the R package drc [107]. Differential response between U87DK and U87H cells were assessed using ANOVA on the fitted parameters.

To eliminate batch effects between plates and experiments performed on different days, a linear mixed-effects model was fitted to the viability measurements from the vehicle control wells on each plate: signal = grand mean+cell line+day of the experiment+plate+residual error, where cell line was classified as a fixed effect term and day of experiment and plate were classified as random effect terms. Model fitting was performed by the R lme4 package [108]. The viability measurements from treatment wells were normalized by subtracting the random effect estimates from all raw signal values before calculating the relative viabilities and fitting the dose-response curves.

### Applying the prize-collecting Steiner tree method

#### Formulation

We used the Goemans-Williamson formulation of the PCST problem. Given an undirected graph 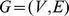 where nodes 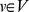 are associated with penalties 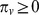 and edges 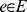 are associated with costs 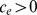, we aim to find a subtree 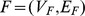 of 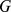 that minimizes the objective function
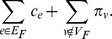
Nodes that have positive penalty values are called “termini”. For our application, the nodes and edges were obtained from protein-protein interaction network datasets (see below). Protein nodes to which experimental data could be mapped received positive penalty values (and therefore they were termini) and other nodes received zero penalties. The cost on edges was inversely related to the confidence on each interaction based on available evidence (see below) so that high confidence edges had lower costs and therefore would be preferentially selected to be in the solution. We further introduced a scaling parameter 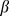 to balance the penalties paid to exclude nodes with experimental observations and the costs of including edges to connect these nodes:
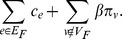
 We solved this optimization problem using the branch-and-cut approach [109] implemented in the dhea-code software program that called the ILOG CPLEX linear programming solver version 12.1 (IBM).

#### Generating solutions that are not trees

To allow the possibility of multiple alternative pathways that may connect the data together despite weaker interaction evidence, we report a composite network (Figure 2) that represents the union of multiple suboptimal solutions. Each suboptimal solution was found by solving the original PCST problem with the additional constraints that 15 percent of the nodes must be different from the original optimal solution (so each suboptimal solution can have a different set of these nodes). The composite network is the union of the nodes and edges from the optimal solution and the nodes and edges from 10 such sub-optimal solutions.

#### Interaction graph and edge cost

We ran PSCIQUIC query [44] to retrieve all the interactions in iRefIndex version 8 [38] for human (species ID 9606). With the PSISCORE Java API [44] each interaction is then scored by the Miscore scoring scheme, which takes into account the number of publications, the experimental method for which the interaction was detected, and the type of the interaction. Since the confidence score 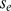 produced by this scheme is log likelihood-based, the cost on each edge for input into the PCST is
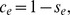
so that minimization on the sum of edge costs can be interpreted as maximizing the product of likelihood. We removed the ubiquitin protein and its connections from the interactome to prevent the solutions being skewed by its high degree of connectivity.

#### Node penalties

We defined two kinds of penalties for proteins in the interaction graph. At the signaling level, we used fold changes in the phosphoproteomics mass-spectrometry (MS) data, and at the level of transcriptional regulation, we used a t-statistic derived from DNase-Seq and mRNA expression data representing our confidence that the transcription factor binds regulatory sites and influences expression. These penalties are described in detail below.

Quantitative phosphotyrosine proteomic data on the U87H and U87DK cells were published previously [37]. Phosphorylated peptide sequences from MS/MS data were matched to the human protein sequences provided in Swiss-Prot database using peptide BLAST program blastp with the following parameters recommended for matching short amino acid sequences: exception value 20000, do not filter low complexity regions, gap opening cost 9, gap extension cost 1, protein scoring matrix PAM30, word size 2, multiple hits window size 40 (-p blastp -e 200000 -F F -G 9 -E 1 -M PAM30 -W 2 -A 40). 100 alignments were requested for each peptide in BLAST XML format report (-b 100 -m 7), which were parsed by the Bio.Blast module in BioPython. Proteins that contained perfect alignment to a peptide sequence received a positive penalty value that was proportional to the absolute value of log-fold change in phosphorylation between the U87H and U87DK cells. If one peptide sequence was aligned to multiple proteins in Swiss-Prot, all these proteins received the same penalty value. If multiple phosphorylated peptide sequences were perfectly aligned to one protein, the maximum fold change in phosphorylation of these peptides was used to calculate the penalty value for this protein.

We derived the penalty values for transcription factors in the protein interaction network from the inferred activity of these transcription factors in inducing changes of mRNA expression. Specifically, we used a regression method to find the correlation between the differential mRNA expression and the sequence specific transcription factor binding motifs in nearby differentially accessible chromatin regions. Filtering the limma analysis results by a maximum p-value of 0.001 adjusted by the Benjamini and Hochberg method [110] gave 2,040 probe sets differentially expressed between U87DK and U87H cells. These probe sets were mapped to 1,623 genes using annotation from the Ensembl Project release 54 (http://may2009.archive.ensembl.org and [111]). From the DNase-Seq data of U87DK and U87H cells, we found genomic regions that were differentially hypersensitive between these two cell lines, i.e., enriched for reads in either U87DK or U87H relative to the other condition, by using the peak caller MACS [112] version 1.4.0beta. For each cell line, aligned reads from the two biological replicates were concatenated. With the U87H read file as the treatment parameter and U87DK read file as the control parameter, a p-value cutoff of 1E-06 and also calling subpeaks, 7,760 peaks, further divided into 13,141 subpeaks, were identified to be more hypersensitive in U87H cells than in U87DK cells. By reversing the treatment and control read files, 5,047 peaks, divided into 9,683 subpeaks, were identified to be more hypersensitive in U87DK cells than in U87H cells. Since functional hypersensitive regions are expected to regulate expression of nearby genes, we evaluated the differential hypersensitive regions identified by MACS and alternative methods based on edgeR [113] for enrichment of nearby differentially expressed genes and obtained similar performance (Figure S4E). Each of the subpeak summits from MACS was mapped to the Ensembl 54 annotated human transcripts that have transcription start sites within 40 kb of the summit, using functionalities in the ChIPpeakAnno package [114] in BioConductor. Sequences from 100 bp upstream and downstream of the subpeak summits were retrieved and the transcription factor affinity scores [25] were computed for the 572 good quality matrices in release 2009.1 of the TRANSFAC database [115]. We then applied the regression procedure for each motif separately, using the affinity scores of the motif in the differential hypersensitive regions and the fold changes in mRNA expression of genes within 40 kb of the differential hypersensitive regions. Let 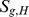 be the set of sequences whose summits are mapped to the gene transcript 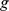 in U87H cells, 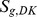 be the set of sequences whose summits are mapped to 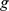 in U87DK cells. Let 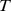 be the set of TRANSFAC matrices and 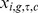 be the affinity score for matrix 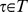 on the 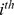 sequence in 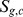 that is mapped to gene 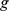 in condition 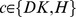. The affinity score of transcription factor matrix 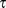 for gene transcript 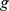 is
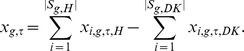
Let 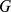 be the set of differential expressed genes described previously and 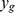 be the log base 2 fold change in expression of transcript 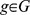 comparing U87H and U87DK cells. For each matrix 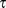 we fit the differential expression of 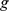 and the affinity score by a univariate linear model:
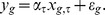
We selected the matrices for which the coefficient of the linear regression was significantly different from zero by a p-value threshold of 0.01 after Bonferroni correction. We used the t-statistic values of the regression coefficients as the penalties on proteins that were assigned to these binding matrices according to TRANSFAC [24], [26], [116]. In cases where one binding matrix corresponded to multiple transcription factors (as commonly found in transcription factor families), all these transcription factors received the same penalty value. Overall, this resulted in 185 significant motifs mapped to 297 proteins in the interactome, capturing 563 differential expressed genes within 40 kb of the differential hypersensitive regions out of the 1,623 differential expressed genes. We explored alternative approaches for defining penalties on the transcription factors, which used edgeR instead of MACS for identifying differential hypersensitive regions and regression with respect to differential DNaseI signal instead of differential expression of nearby genes (Figure S4). The penalty values and the subsequent PCST solution did not change significantly.

We considered the magnitude of node penalties defined above as a measure for the activities of the protein termini revealed by the experimental data. Since the PCST algorithm minimizes penalty values on termini excluded from the final network, it will preferentially include proteins with larger penalty values, i.e., bigger fold changes in phosphorylation or stronger association of the transcription factor motif to changes in target gene expression. In the absence of formal statistical model for the likelihood of these data, we tested the sensitivity of the PCST solution in Figure 2 to different levels of noise artificially introduced into the node penalties (Figure S5) and concluded that the solution is robust and by multiple statistics could easily tolerate noise level up to 20%.

### Choosing the parameter 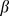


The parameter 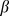 controls the size of the PCST network by balancing the edge costs and node penalties. We would like the solution network to be small and connect a large number of termini by a small number of Steiner nodes. Therefore, we defined the “efficiency ratio” of a PCST solution as the ratio of included terminal nodes to Steiner nodes and selected a value of 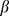 such that the solution network is small and has a good efficiency ratio. Specifically, we ran the algorithm with a wide range of 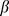 values and computed the efficiency ratios for the solutions (Figure S6). We found that the efficiency ratio was relatively stable for 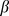 values between 40 and 100, although the rate of increase of this ratio was slightly larger between 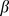 of 50 and 60 (so increasing network size is more efficient in connecting the termini) and the network solution is intermediate in size. The PCST presented in Figure 2 was from 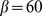.

### EGFRvIII mouse GBM xenograft phosphoproteomic data

A complete list of tyrosine phosphorylated peptides was downloaded from the supplementary data of [61], which were collected from mouse xenograft samples established from patient surgical specimens. Among the eight samples, two express wild-type EGFR at normal level, three express amplified level of wild-type EGFR, and three express amplified level of EGFRvIII. For each phosphorylated peptide, Student's t-test was used to compare the three samples with amplified EGFRvIII to the two samples with normal level of wild-type EGFR, and those peptides with p-value less than 0.05 were considered differentially phosphorylated in response to EGFRvIII.

### Scoring interactome nodes with respect to the PCST solution

For each node in the interactome, either inside or outside of the PCST solution, we found the edges connecting this node to the nodes in the PCST solution and summed up the confidence scores on these edges (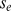 described above). Since the score on each edge is a log-likelihood confidence score of this interaction, a large value of this sum means this node has more high confidence interactions with the nodes in the PCST solution. We then ranked all the nodes in the interactome by this score.

### TCGA GBM exon array processing and enrichment analysis of transcription factor targets

Level 3 exon array data of the TCGA GBM project [53], [62] were downloaded from the TCGA data portal (https://tcga-data.nci.nih.gov/tcga/). The U87 cell line is reported to contain wild-type p53 and deletion of p16 [117], which match the mutation status of 99 patients as reported in the cBio Portal [118]. The EGFRvIII status of these 99 patients were determined by testing for significantly lower expression of the exon array probe sets that map to exon 2–7 of the EGFR gene (p<0.05 by Wilcoxon rank-sum test). 19 such samples were found and were thus labeled as EGFRVIII. Gene level expression values from the exon array were then analyzed for differential expression between the EGFRvIII and non-EGFRvIII samples by limma [103]. Hierarchical clustering of the differentially expressed genes (p-value<0.01) show that the 17 of the 19 patients with EGFRvIII mutation were grouped together among the major clusters (Figure S2).

Separately, for each transcription factor that has a motif in TRANSFAC, we used the MATCH program [119] to score for matches to the motif in the DNaseI hypersensitive regions that are more hypersensitive in the U87H cells than in the U87DK cells. This resulted in a set of factors for the U87H cells (U87H TF). The set of U87DK TF were found similarly from regions with higher hypersensitivity in the U87DK cells. If there was a motif match within 40 kb of the transcription start site of a gene, this gene was considered a target of the TF. Then for each TF, we computed the minimum hypergeometric (mHG) p-value [63] for testing the enrichment of its target genes in the list of all genes in the TCGA expression dataset ranked by log fold change between patients with the EGFRvIII mutation and those without. To determine the role of transcription factors in up-regulation, we calculated the mHG p-value of the genes ranked from highest changes in expression in EGFRvIII patients to lowest changes in expression. To determine the role of genes in down-regulation, we reversed the order of genes. P-values in Figure 3E and Figure 3F were calculated using Student's t-test.

### Scoring p300 interactors

We searched for sequence motifs that could predict which of the regions that were more hypersensitive in U87H than in U87DK cells were bound by p300. Differentially hypersensitive regions overlapping with p300 bound regions in U87H from ChIP-Seq were considered positive and the rest were negative. We then computed matches to the sequence motifs in the TRANSFAC vertebrate non-redundant set in all the positive and negative regions. The motif match scores were used in a feature selection procedure by Wilcoxon test in five-fold cross validation to rank these motifs by their ability to classify the positive and negative regions. To compute the significance of the p300 interactors inside the PCST to those outside of the PCST, the top 20 motifs were selected in each iteration of the cross-validation. Those correspond to proteins that interact with p300 were recorded and enrichment p-values were computed by Fisher exact test.

### Accession numbers

The raw data for ChIP-Seq, DNase-Seq and microarray experiments have been submitted to GEO under accession number GSE36902.

## Supporting Information

Figure S1 Correlation is poor between changes in tyrosine phosphorylation on proteins and changes in transcript level of the corresponding mRNA observed in the U87DK and U87H cells.
(PDF)

Figure S2 Hierarchical clustering by exon array gene expression of TCGA GBM patient samples with wild-type p53 and deleted p16. The set of differentially expressed genes (limma p-value<0.01) were the input to the clustering. Each row in the heatmap is a gene and each column is one sample. EGFRvIII status of a sample is indicated by the color code at the top of the heatmap.
(PDF)

Figure S3 Distribution of GI50 values (the concentration that inhibit growth by 50%) of the NCI60 panel of cell lines for compounds that were used in this study and also tested in the In Vitro Cell Line Screening Project (IVCLSP) of the Developmental Therapeutics Program at the National Cancer Institute. Paclitaxel and rapamycin were tested more than once in the IVCLSP at the indicated maximum concentrations. The vertical dashed lines mark the concentrations of the compounds for the viability data presented in Figure 4A.
(PDF)

Figure S4 Assessment of multiple methods for identification of differential DNaseI hypersensitive regions. A. Scatter plot of log2 read counts from the U87DK and U87H cells inside the DNaseI hypersensitive regions that were called by MACS to be differentially hypersensitive (p<1E-06) and otherwise. B Same scatter plot as in A, but the differential hypersensitive regions were determined by the edgeR method [113] included in the R DiffBind package [122] (FDR<0.1). C. Same scatter plots as in A and B but each differential hypersensitive region is color coded by whether it was reported by MACS alone, edgeR alone or both. The hypersensitive regions not reported to be different between the two conditions were omitted in this plot. D. Cumulative distribution of log2 read counts from the less hypersensitive condition inside the differential hypersensitive regions, grouped by whether that region is called differentially hypersensitive by MACS alone, edgeR alone, or both. MACS does not have strong bias for regions that are closed (containing lower read counts) in one condition. E. Enrichment of differentially expressed genes within 40 kb of the differential hypersensitive regions reported by MACS and edgeR. Enrichment p-values shown on top of each method were computed by Fisher exact test relative to whole genome background. F. Scatter plot and correlation coefficients among the TF termini penalty values computed by (i) regression of differential expression to motif affinity score in the nearby differential hypersensitive regions called by MACS (“MACS expr”); (ii) regression of differential hypersensitivity to motif affinity score inside the differential hypersensitive regions called by MACS (“MACS HS”); (iii) same regression as in (ii) but with the differential hypersensitive regions determined by edgeR (“edgeR HS”). Lower left panels are scatter plots of penalty values derived from methods in the corresponding row and column. Upper right panels are Pearson correlation coefficients between the penalty values. G. Left: overlap of the set of TF termini from the three regression approaches described in F. Middle: overlap of PCST solutions using TF termini from the regression approaches in F and the pY termini. Right: overlap of the PCST solutions as in the middle panel, with the pY termini excluded.
(PDF)

Figure S5 The PCST solution was robust to noise in node penalties and edge costs. The node penalties used to find the U87 PCST network were multiplied by a random factor that is normally distributed with mean 1 and a specific level of standard deviation (0.05, 0.1, 0.2, 0.25, 0.5) and a new PCST network was found for this set of randomized node penalties. For each level of the standard deviation, 100 such PCST solutions were obtained and compared to the original PCST. Randomization on edge costs were performed similarly. A. The mean and standard error for the percentage of nodes in the original PCST that appear in the randomized PCST. 80% of the nodes in the original PCST still appear in the randomization solutions even at 50% noise. B. The mean and standard error of the Jaccard Index (size of intersection divided by size of union) comparing the PCST solutions from randomization to the original PCST. The solution was more sensitive to changes in edge cost values but still showed Jaccard index about 0.75 at 20% noise. At much higher noise levels, the randomization solutions went through other nodes in the interactome, indicating that optimization on the edge costs indeed controlled the nodes included in the PCST solution.
(PDF)

Figure S6 Plot of efficiency ratio (number of terminal nodes divided by number of Steiner nodes) for different sizes of the PCST solutions obtained from the indicated β values. The efficiency ratio was relatively stable for β values between 40 and 100. Within this range, the rate of increase of efficiency ratio with respect to network size is the slightly largest between β of 50 and 60. The PCST presented in Figure 2 was from β of 60.
(PDF)

Table S1 List of differentially expressed genes between the U87H and U87DK cells. Columns are: Affymetrix probe ID, official gene symbol, gene name, UniGene ID, (remaining columns are output from limma) log base 2 fold change of the probe in U87H cells relative to U87DK cells, average log base 2 expression of the probe over all arrays, moderated t-statistic, raw p-value, adjusted p-value, log-odds that the gene is differentially expressed.
(TXT)

Table S2 Significantly enriched motifs in the proximal promoter of genes differentially expressed between U87H and U87DK. The differentially expressed genes were analyzed by the motif enrichment function of Expander [123] for enriched motifs in -1000 to +200 bp from the transcription start site using vertebrate TRANSFAC motifs with a corrected p-value threshold of 0.05. Five sets of genes were analyzed: HToDK_DEG (genes differentially expressed between U87H and U87DK with adjusted p-value lower than 1E-03), HToDK_DEG_DOWN (genes in HToDK_DEG and down-regulated in U87H relative to U87DK), HToDK_DEG_UP (genes in HToDK_DEG and up-regulated in U87H relative to U87DK), HToDK_DEG_FC2 (genes differentially expressed between U87H and U87DK with adjusted p-value less than 1E-03 and fold change greater than 2), HToDK_DEG_FC2_UP (genes in HToDK_DEG_FC2 and up-regulated in U87H relative to U87DK), and HToDK_DEG_FC2_DOWN (genes in HToDK_DEG_FC2 and down-regulated in U87H relative to U87DK). No motifs passed the significance threshold for the set HToDK_DEG_UP and HToDK_DEG_FC2_UP.
(TXT)

Table S3 Illumina sequencing statistics of the DNase-Seq and p300 ChIP-Seq samples. Aligned unique: reads that are uniquely aligned to the genome. Aligned repeat: reads that are aligned to more than one locations in the genome. Aligned none: reads that are not aligned to any location in the genome.
(TXT)

Table S4 Ranking of motifs by how well the motif scores can classify hypersensitive regions that overlap with p300 bound regions from those that do not overlap with p300 bound regions. For each iteration in five-fold cross-validation, the top 20 motifs out of a total of 151 are listed. Columns are: iteration number in five-fold cross-validation, rank of motif, TRANSFAC ID of motif, whether the motif is mapped to a p300 interacting protein inside the PCST (1 is yes, 0 is no), whether the motif is mapped to a p300 interacting protein outside of the PCST (1 is yes, 0 is no).
(TXT)

Table S5 Enriched GO categories of p300 bound genes in GM12878 cells and HepG2 cells. Files containing p300 bound peaks detected by ChIP-Seq were downloaded from the UCSC Genome Browser and represented the ENCODE Jan 2010 Freeze for GM12878 and July 2009 Freeze for HepG2. Mapping peaks to genes and GO analysis were performed using the same parameters as for the U87H p300 ChIP-Seq dataset.
(TXT)
