# Supplementary figures and images for: Linking Proteomic and Transcriptional Data through the Interactome and Epigenome Reveals a Map of Oncogene-induced Signaling
Source: PLoS Comput Biol. 2013 Feb 7;9(2):e1002887. doi: 10.1371/journal.pcbi.1002887 (PMC3567149; doi:10.1371/journal.pcbi.1002887)

Correlation = 0.0914287405277691

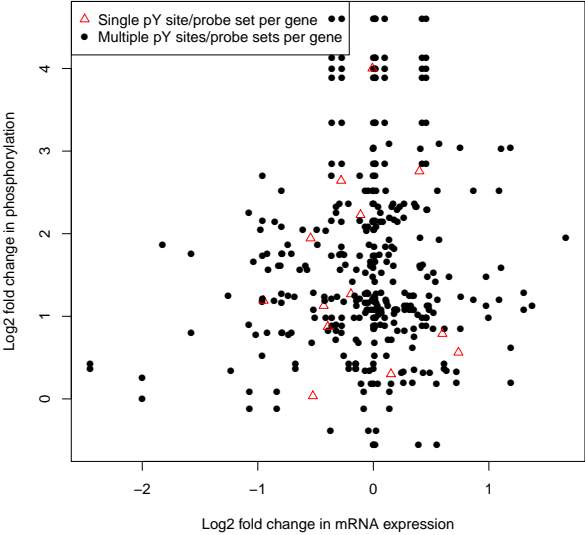

Supplement: Figure S1 — Correlation is poor between changes in tyrosine phosphorylation on proteins and changes in transcript level of the corresponding mRNA observed in the U87DK and U87H cells. (PDF) [file pcbi.1002887.s001.pdf]

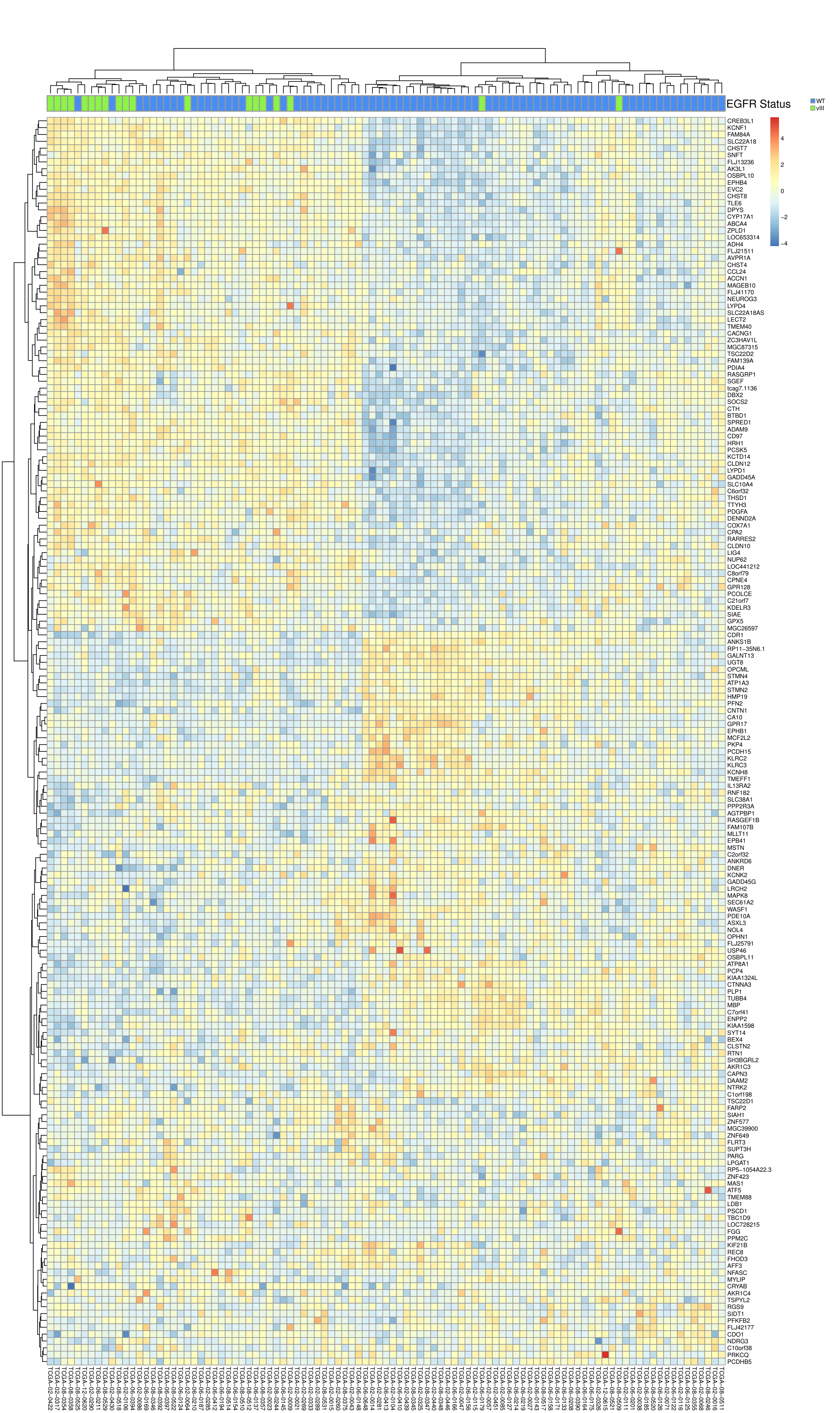

Supplement: Figure S2 — Hierarchical clustering by exon array gene expression of TCGA GBM patient samples with wild-type p53 and deleted p16. The set of differentially expressed genes (limma p-value<0.01) were the input to the clustering. Each row in the heatmap is a gene and each column is one sample. EGFRvIII status of a sample is indicated by the color code at the top of the heatmap. (PDF) [file pcbi.1002887.s002.pdf]

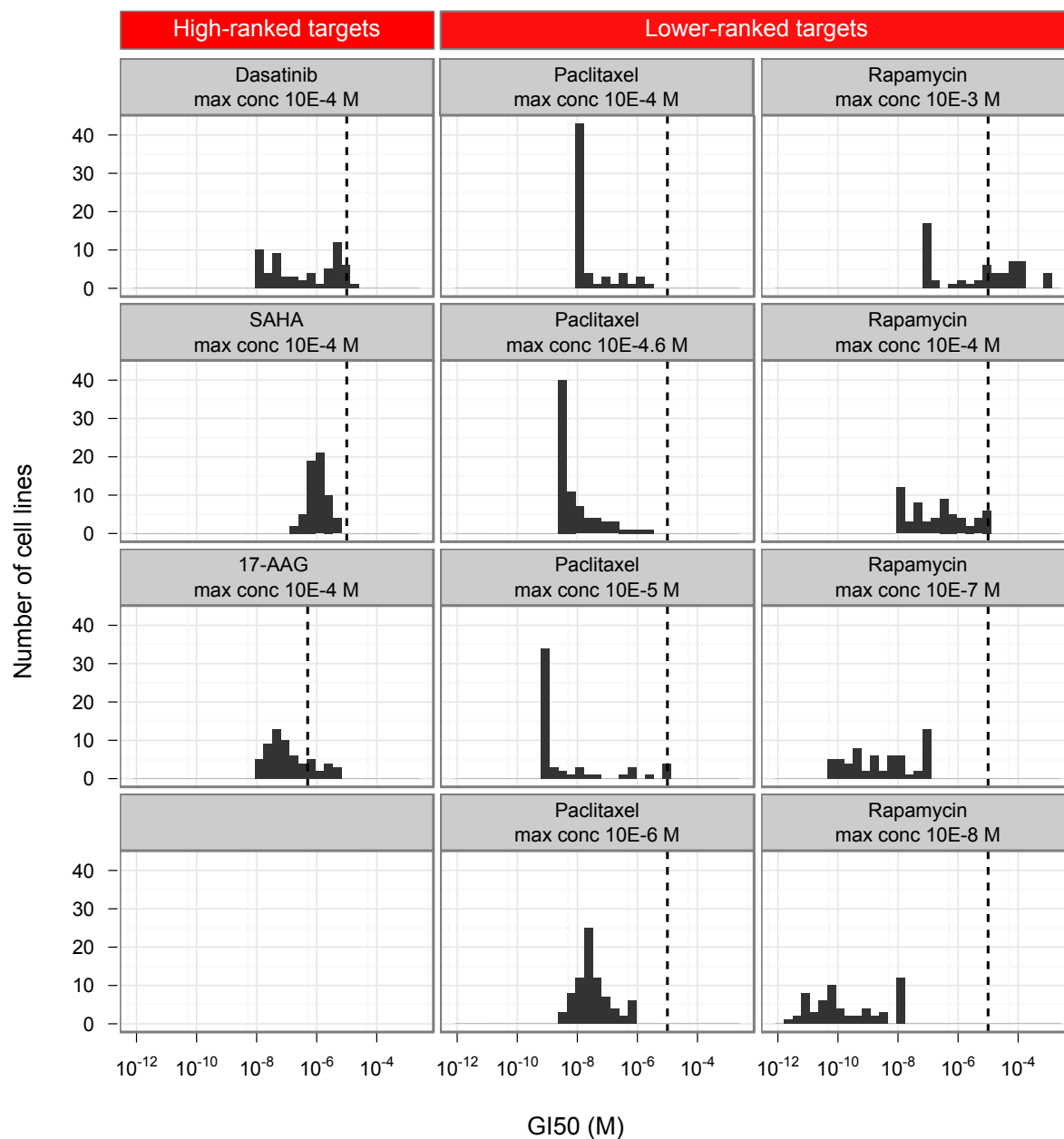

Supplement: Figure S3 — Distribution of GI50 values (the concentration that inhibit growth by 50%) of the NCI60 panel of cell lines for compounds that were used in this study and also tested in the In Vitro Cell Line Screening Project (IVCLSP) of the Developmental Therapeutics Program at the National Cancer Institute. Paclitaxel and rapamycin were tested more than once in the IVCLSP at the indicated maximum concentrations. The vertical dashed lines mark the concentrations of the compounds for the viability data presented in Figure 4A. (PDF) [file pcbi.1002887.s003.pdf]

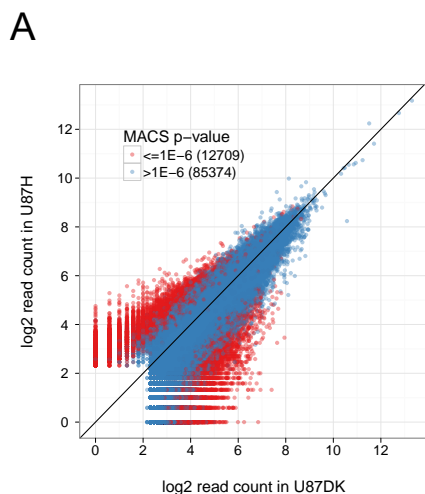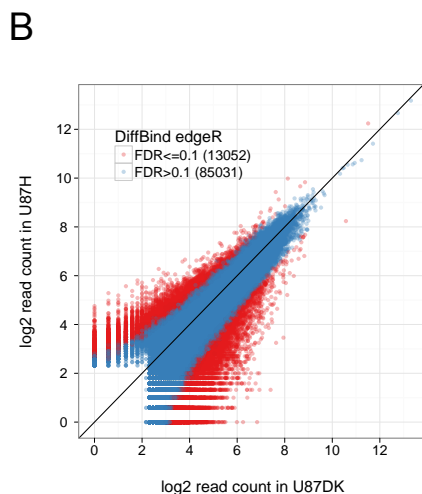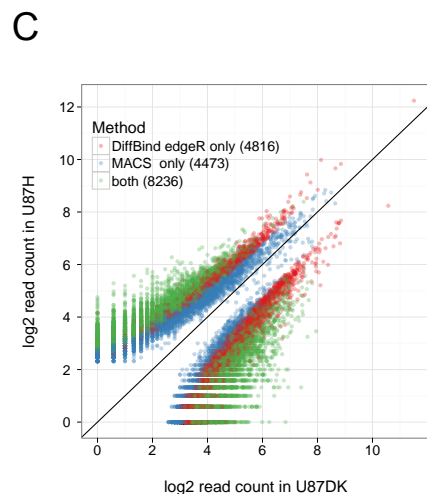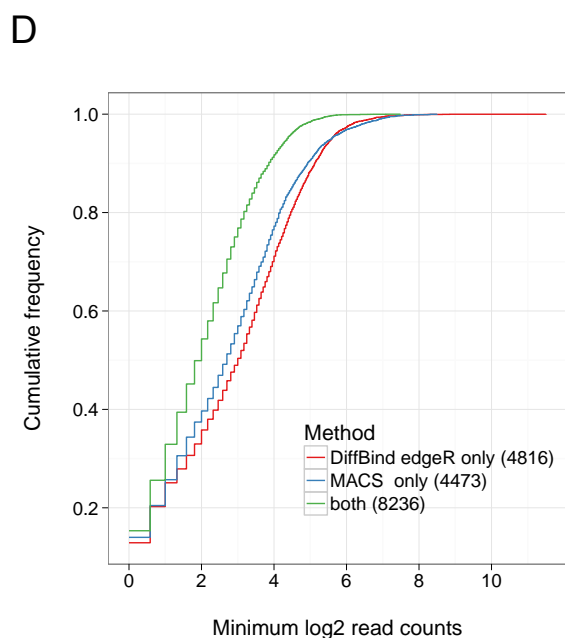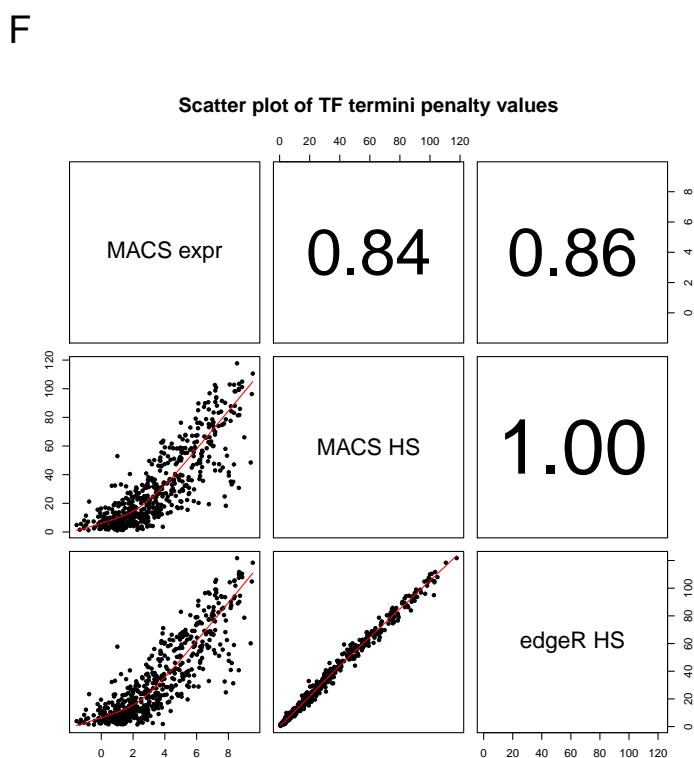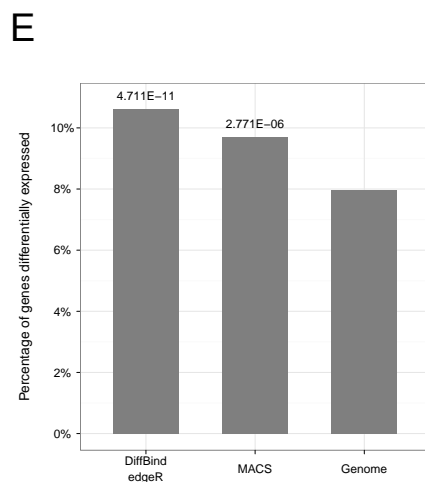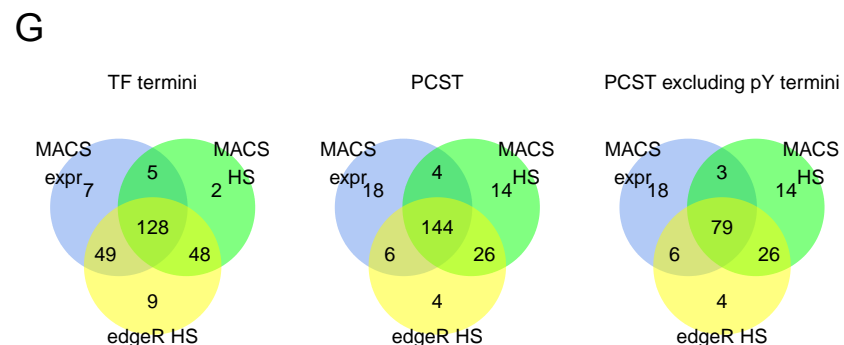

Supplement: Figure S4 — Assessment of multiple methods for identification of differential DNaseI hypersensitive regions. A. Scatter plot of log2 read counts from the U87DK and U87H cells inside the DNaseI hypersensitive regions that were called by MACS to be differentially hypersensitive (p<1E-06) and otherwise. B Same scatter plot as in A, but the differential hypersensitive regions were determined by the edgeR method [113] included in the R DiffBind package [122] (FDR<0.1). C. Same scatter plots as in A and B but each differential hypersensitive region is color coded by whether it was reported by MACS alone, edgeR alone or both. The hypersensitive regions not reported to be different between the two conditions were omitted in this plot. D. Cumulative distribution of log2 read counts from the less hypersensitive condition inside the differential hypersensitive regions, grouped by whether that region is called differentially hypersensitive by MACS alone, edgeR alone, or both. MACS does not have strong bias for regions that are closed (containing lower read counts) in one condition. E. Enrichment of differentially expressed genes within 40 kb of the differential hypersensitive regions reported by MACS and edgeR. Enrichment p-values shown on top of each method were computed by Fisher exact test relative to whole genome background. F. Scatter plot and correlation coefficients among the TF termini penalty values computed by (i) regression of differential expression to motif affinity score in the nearby differential hypersensitive regions called by MACS (“MACS expr”); (ii) regression of differential hypersensitivity to motif affinity score inside the differential hypersensitive regions called by MACS (“MACS HS”); (iii) same regression as in (ii) but with the differential hypersensitive regions determined by edgeR (“edgeR HS”). Lower left panels are scatter plots of penalty values derived from methods in the corresponding row and column. Upper right panels are Pearson correlation coefficients be [file pcbi.1002887.s004.pdf]

A

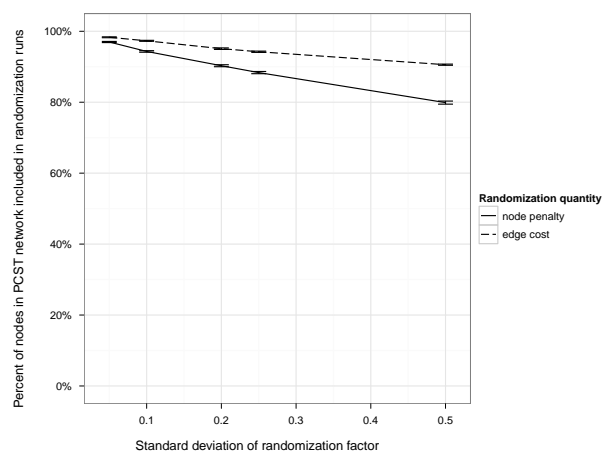

B

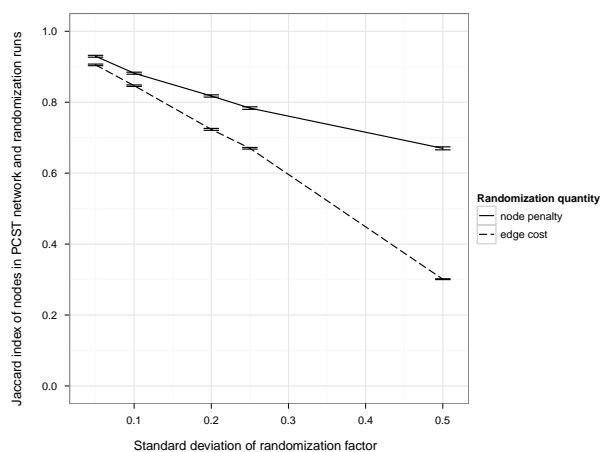

Supplement: Figure S5 — The PCST solution was robust to noise in node penalties and edge costs. The node penalties used to find the U87 PCST network were multiplied by a random factor that is normally distributed with mean 1 and a specific level of standard deviation (0.05, 0.1, 0.2, 0.25, 0.5) and a new PCST network was found for this set of randomized node penalties. For each level of the standard deviation, 100 such PCST solutions were obtained and compared to the original PCST. Randomization on edge costs were performed similarly. A. The mean and standard error for the percentage of nodes in the original PCST that appear in the randomized PCST. 80% of the nodes in the original PCST still appear in the randomization solutions even at 50% noise. B. The mean and standard error of the Jaccard Index (size of intersection divided by size of union) comparing the PCST solutions from randomization to the original PCST. The solution was more sensitive to changes in edge cost values but still showed Jaccard index about 0.75 at 20% noise. At much higher noise levels, the randomization solutions went through other nodes in the interactome, indicating that optimization on the edge costs indeed controlled the nodes included in the PCST solution. (PDF) [file pcbi.1002887.s005.pdf]

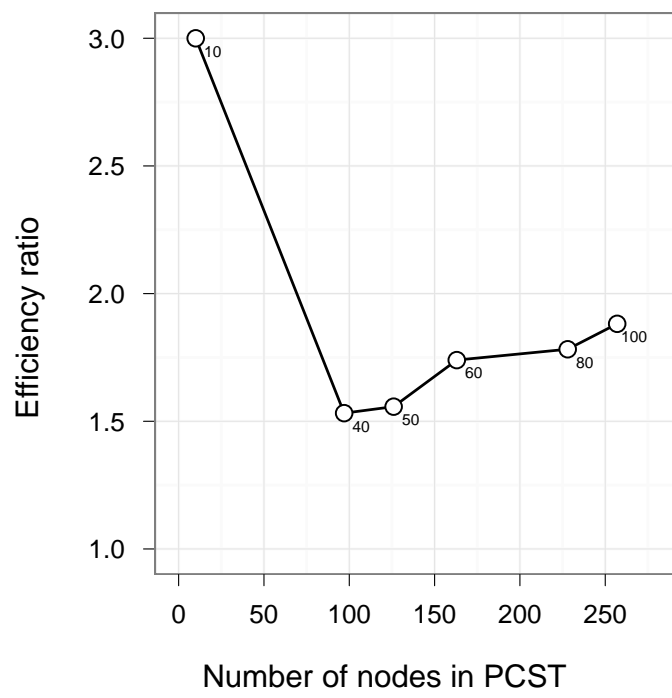

Supplement: Figure S6 — Plot of efficiency ratio (number of terminal nodes divided by number of Steiner nodes) for different sizes of the PCST solutions obtained from the indicated β values. The efficiency ratio was relatively stable for β values between 40 and 100. Within this range, the rate of increase of efficiency ratio with respect to network size is the slightly largest between β of 50 and 60. The PCST presented in Figure 2 was from β of 60. (PDF) [file pcbi.1002887.s006.pdf]
